# Myeloma cells can corrupt senescent mesenchymal stromal cells and impair their anti-tumor activity

**DOI:** 10.18632/oncotarget.5430

**Published:** 2015-10-16

**Authors:** Servet Özcan, Nicola Alessio, Mustafa Burak Acar, Güler Toprak, Zeynep Burcin Gönen, Gianfranco Peluso, Umberto Galderisi

**Affiliations:** ^1^ Genome and Stem Cell Center (GENKOK), Erciyes University, Kayseri, Turkey; ^2^ Department of Experimental Medicine, Biotechnology and Molecular Biology Section, Second University of Naples, Naples, Italy; ^3^ Sbarro Institute for Cancer Research and Molecular Medicine, Center for Biotechnology, Temple University, Philadelphia, PA, USA; ^4^ Institute of Bioscience and Bioresources, CNR, Naples, Italy; ^5^ Department of Biology, Faculty of Sciences, Erciyes University, Kayseri, Turkey; ^6^ Graduate School of Natural and Applied Sciences, Erciyes University, Kayseri, Turkey

**Keywords:** Gerotarget, mesenchymal stem cells, senescence, secretome, apoptosis, cancer

## Abstract

Senescent cells secrete several molecules that help to prevent the progression of cancer. However, cancer cells can also misuse these secreted elements to survive and grow. Since the molecular and functional bases of these different elements remain poorly understood, we analyzed the effect of senescent mesenchymal stromal cell (MSC) secretome on the biology of ARH-77 myeloma cells. In addition to differentiating in mesodermal derivatives, MSCs have sustained interest among researchers by supporting hematopoiesis, contributing to tissue homeostasis, and modulating inflammatory response, all activities accomplished primarily by the secretion of cytokines and growth factors. Moreover, senescence profoundly affects the composition of MSC secretome. In this study, we induced MSC senescence by oxidative stress, DNA damage, and replicative exhaustion. While the first two are considered to induce acute senescence, extensive proliferation triggers replicative (i.e., chronic) senescence. We cultivated cancer cells in the presence of acute and chronic senescent MSC-conditioned media and evaluated their proliferation, DNA damage, apoptosis, and senescence. Our findings revealed that senescent secretomes induced apoptosis or senescence, if not both, to different extents. This anti-tumor activity became heavily impaired when secretomes were collected from senescent cells previously in contact (i.e., primed) with cancer cells. Our analysis of senescent MSC secretomes with LC-MS/MS followed by Gene Ontology classification further indicated that priming with cancer profoundly affected secretome composition by abrogating the production of pro-senescent and apoptotic factors. We thus showed for the first time that compared with cancer-primed MSCs, naïve senescent MSCs can exert different effects on tumor progression.

## INTRODUCTION

Confronted with endogenous and exogenous stresses, cells may respond by becoming senescent. Genomic stressors such as the shortening of chromosome telomeres, non-telomeric DNA damage, excessive mitogenic signals, and non-genotoxic stress such as perturbations to chromatin organization are all considered to be damaging events. In response, senescence bars the onset of cancer by blocking the proliferation of transformed cells. It may also contribute to other biological processes such as development, tissue repair, and aging [1, 2].

Though cellular senescence has been deemed a unique intracellular event triggered by the activation of cytoplasmic signaling circuitry, it is now evident that senescent cells secrete dozens of molecules, for which the term *senescence-associated secretory phenotype* (SASP) has been proposed. The secreted factors contribute to cellular proliferative arrest through autocrine/paracrine pathways [3–5].

SASP released by senescent cells can signal danger that sensitizes normal surrounding cells to senesce, thereby improve the likelihood that damaged cells enter senescence. Senescent secretome contains cytokines that attract and activate cells of the immune system, which can in turn dispose of the senescent cells. However, SASP can also exert deleterious effects, for the presence of senescent cells in tissue can contribute to impairing its functions by triggering the senescence of healthy cells as well. The secretome of senescent cells can also contain numerous inflammatory cytokines, growth factors, and proteases that can render the tissue microenvironment favorable for tumor growth, since some tumor cells misuse SASP for their own growth [2, 5, 6]. The secretome of senescent cells can also facilitate angiogenesis and epithelial–mesenchymal transition, as well as promote the proliferation of cancer cells [3, 6–9].

For the above reasons, the study of SASP produced by mesenchymal stromal cells (MSCs) is of great interest. MSCs contain a subpopulation of stem cells able to differentiate in mesodermal derivatives (e.g., adipocytes, chondrocytes, osteocytes) and can also contribute to the homeostatic maintenance of several organs [10, 11]. MSCs execute their multiple functions by secreting a range of cytokines and growth factors [12]. Senescence greatly alters the composition of this secretome, namely by changing levels of proteins involved in extracellular matrix (ECM) remodeling and in key regulators of insulin growth factor-signaling pathways. Both processes are known to contribute to the initiation of senescence and cancer [5, 13].

To date, however, no studies have reported the effects of senescent MSC secretome upon the biology of cancer cells. In this sense, the importance of our findings rests in our observation that healthy MSCs have been associated with tumor progression. MSC secretome can contribute to tumor growth in several ways: by promoting angiogenesis, creating a niche to support cancer stem cells survival, modulating the organism's immune response against cancer cells, and by promoting the formation of metastasis [14]. To contribute to these findings, we investigated the effects of senescent MSC secretome upon the *in vitro* behavior of ARH-77 cells, which constitute a useful model of myeloma.

Different genotoxic stressors induce phenotypically different cellular senescent states with features both common and specific, though primarily concerning expressed genes and secreted factors. Furthermore, acquiring a senescent phenotype constitutes a progressive process that is reversible before transient cell-cycle arrest becomes stable [1, 2].

In this study, we induced MSC senescence by using three different mechanisms: oxidative stress, DNA damage, and replicative exhaustion. While the first two mechanisms are considered to induce acute senescence, extensive proliferation triggers replicative (i.e., chronic) senescence [1, 2].

We cultivated cancer cells in the presence of acute and chronic senescent MSC-conditioned media (CM) and evaluated their proliferation, DNA damage, apoptosis, and senescence. Our findings indicated that senescent secretomes induced apoptosis or senescence, if not both, to different extents. However, this anti-tumor activity became heavily impaired when secretomes were collected from senescent cells previously in contact (i.e., primed) with cancer cells.

## RESULTS

### Naïve senescent MSC secretomes reduced the cycling capacity of ARH-77 and promoted senescence and apoptosis

**Figure 1:**
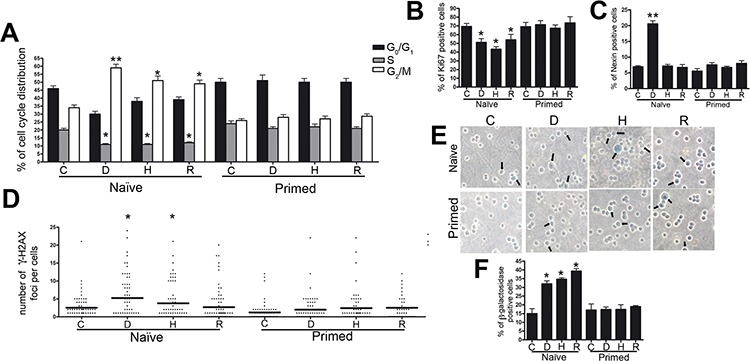
Biological effects of senescent MSC secretomes on myeloma cells **Panel A.** Histograms depict the cell cycle profiles of ARH-77 cells incubated with control (C) or senescent secretomes; D, H, and R indicate groups treated with secretomes obtained from doxorubicin- or H_2_0_2_-treated and replicatively senescent MSCs, respectively. **Panel B.** Percentage of cycling (Ki-67+) ARH-77 cells in the presence of different secretomes. **Panel C.** Percentage of apoptotic ARH-77 cells following incubation with control or senescent secretomes. **Panel D.** Graph purporting the degree of H2AX phosphorylation evaluated by counting the number of gamma-H2AX immunofluorescent foci per cell; each foci number was determined for 200 cells; each dot represents an individual cell, while black bars indicate the mean value for each category. **Panel E.** Representative microscopic fields of acid β-galactosidase (blue) in ARH-77 cells; arrows indicate senescent cells. **Panel F.** Graph showing the mean percentage value of senescent cells. All data are shown with standard deviation. For experiments depicted in each panel, we performed one-way analysis of variance followed by Bonferroni tests for multiple comparisons. The null hypothesis assumed no difference between the experimental (D, H, R) and control groups (*p < .05, **p < .01).

Naïve senescent secretomes affected the cell cycle profile of ARH-77 cells (Figure 1A). In particular, replicative and H_2_0_2_ senescent secretomes (Naïve-R and Naïve-H, respectively) induced a significant reduction of cells in the S phase compared with the cell count of the controls. By contrast, senescent secretome doxorubicin (Naïve-D) primarily caused G_2_/M arrest. Consistent with these data, a decline in cycling (Ki-67+) cells was also observed when cultures were incubated with senescent secretomes (Figure 1B).

Annexin V assay showed that unlike all other CM, only Naïve-D induced a significant increase in the percentage of apoptosis in ARH-77 cultures (Figure 1C). These data were confirmed by evaluating active caspase 9 (data not shown). At the same time, all naïve senescent secretomes promoted senescence to high levels in cultures incubated with Naïve-D (Figure 1E and 1F).

Increased senescence and apoptosis induced by treatment with naïve senescent secretomes suggest both that DNA damages subsequent to genotoxic stress were incompletely repaired and that the cells may have persistent foci with damaged DNA (DNA-SCARS). Indeed, in the nuclei of ARH-77 cells treated with Naïve-D and Naïve-H, we detected several phosphorylated H2AX (gamma-H2AX) foci, which can indicate damaged DNA (Figure 1D). In cells incubated with Naïve-R, the foci did not increase compared with those in the control group.

More importantly, this finding suggests that naïve senescent MSC secretomes lack any stimulus for cancer cell growth. This possibility is of great interest, since other findings have indicated that the secretome of senescent cells may promote cancer growth [3, 7–9, 15, 16]. All experiments reported above were also conducted on Kasumi-1 cells derived from a human acute myeloblastic leukemia. Results overlapped those obtained with the ARH-77 cell line (data not shown).

### Cancer-primed senescent MSC secretomes have an impaired ability in controlling cancer growth

To gain further insights into the effect of senescent MSC secretomes upon cancer growth, we investigated another crucial aspect of MSC function: its priming effect on MSCs. Different toll-like receptors can polarize MSCs into two distinct populations that express either pro-inflammatory or suppressive factors [17–19]. In this scenario, senescent cells primed with cancer cells may be forced to produce cytokines and other factors for their survival and growth. In evaluating the effect of cancer-primed senescent MSC secretomes on the *in vitro* behavior of ARH-77 myeloma cells, we observed that priming abrogated the cell cycle of naïve senescent secretomes (Figure 1A). Specifically, primed doxorubicin- and H_2_0_2_-treated senescent secretomes (Primed-D and Primed-H, respectively) induced an increase in S phase compared with the control group. By contrast, primed replicatively senescent secretome (Primed-R) showed a cell cycle profile similar to that of the control cultures (Figure 1A). Notably, the incubation of cancer cells with primed senescent secretomes did not reduce the percentage of cycling cells (Ki-67+) detected with naïve secretomes (Figure 1B). Furthermore, Primed-D did not induce the increase in apoptosis in ARH-77 cultures observed with Naïve-D (Figure 1C). The significant rise in senescence detected with naïve secretomes did not emerge in cancer cultures incubated with primed secretomes. In fact, in all tested conditions, senescence did not differ significantly from that of the control group (Figure 1E–1F).

### Secretome analysis of naïve and primed senescent MSCs

To identify molecules possibly responsible for the anti-tumor properties in naïve senescent secretomes and to examine how priming affects secretome composition, we conducted LC-ESI-MS/MS analyses on peptides from the tryptic digestion of secretome proteins. Using high-resolution MS in a search of the Protein Metrics database, we identified 482, 1,087, and 521 proteins in Naïve-R, Naïve-D, and Naïve-H, respectively (Supplementary File S1). By extension, in our analysis of primed secretomes, we identified 517, 348, and 319 proteins in Primed-R, Primed-D, and Primed-H, respectively (Supplementary File S2).

A preliminary analysis of secretome composition indicated that a fraction of proteins in the secretomes lacked the signal peptide for the classical endoplasmic reticulum–Golgi secretion pathway. It is reasonable to hypothesize that they come from non-classical secretion pathways, including those of exosomes and microvesicles, extracellular vesicles that represent a recently discovered means of intercellular communication for the transfer of membrane-bound and cytosolic proteins, lipids, and RNA among cells [20, 21].

**Figure 2:**
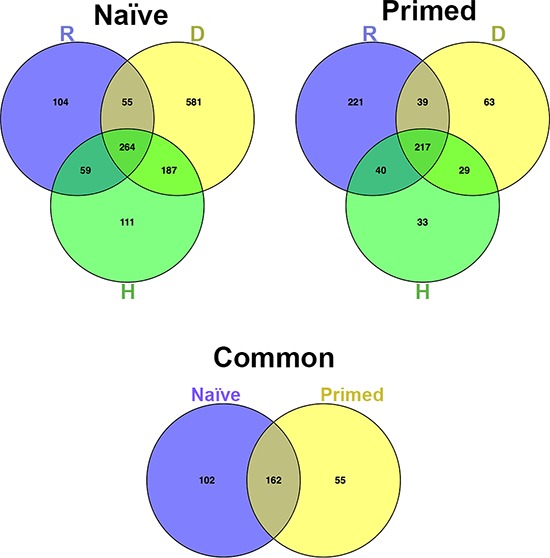
Venn diagram analysis **Top left:** Venn diagram showing common and specific proteins among naïve secretomes obtained from (D) doxorubicin- or (H) H_2_0_2_-treated and (R) senescent replicative MSCs. **Top right:** Comparison of primed secretomes. **Bottom:** Venn diagram comparing the 264 proteins common among naïve secretomes with the 217 proteins common among primed secretomes.

In correlating the secretome profiles with biological phenomena observed, we considered that the anti-tumor effect of naïve secretomes (i.e., cell cycle arrest, apoptosis, and senescence) represented a shared function of the three different senescent types. Accordingly, we focused on proteins common among the three senescent secretomes, since this collated set of molecules should include factors responsible for the shared biological activities. Venn diagram analysis identified 264 proteins shared among Naïve-R, Naïve-D and Naïve-H (Figure 2) and 217 proteins shared among primed secretomes that could be associated with the loss of anti-tumor capacity. To refine our results, we used Venn analysis to determine which of the 264 proteins shared in naïve secretomes were expressed exclusively in naïve cells, as well as which of the 217 proteins present in primed secretomes were expressed only in primed cells. We ultimately identified 102 proteins present exclusively in naïve secretomes and 55 exclusively in primed ones (Figure 2).

Gene Ontology (GO) and network analyses were performed on proteins detected exclusively in either naïve or primed secretomes. Shown in Table 1, results from the Protein Analysis through Evolutionary Relationships (PANTHER) database exhibited striking differences between naïve and primed secretomes, as Supplementary File S3 describes in greater detail. For example, naïve secretomes were enriched for proteins involved in biological functions such as translation, proteolysis, and protein metabolic processes. By contrast, primed secretomes contained proteins involved in morphogenesis and developmental processes. Shown also in Table 1, in the naïve secretome we identified hundreds of proteins associated with cytoskeletal and extracellular matrix remodeling, which Supplementary File S3 describes in greater detail.

**Table 1:** Summary of Gene Ontology analysis performed with GO slims (PANTHER)

GO Slim biological processes			GO Slim molecular functions			Go Slim protein classes		
	Naïve	Primed		Naïve	Primed		Naïve	Primed
**Translation**	◊ M SP SF		**Actin binding**	◊ E		**Ribosomal**	◊ M	
**Proteolysis**	◊ M SP SF		**Cytoskeletal protein binding**	◊ E		**Actin family**	◊ E	◊ E
**Protein metabolic process**	◊ M		**Calcium ion binding**	◊ E		**Cytoskeleton**	◊ E	◊ E
**Primary metabolic process**	◊ M		**Structural molecular actin**	◊ E	◊ E			
**Cellular component morphogenesis**		◊	**Structural constituents cytoskeleton**	◊ E	◊ E			
**Anatomical structural morphology**		◊	**Protein binding**		◊ E			
**Developmental process**		◊						

Ontological terms classified as biological processes, molecular functions, and molecular classes; diamonds indicate the presence of a specific ontological term in either naïve or primed secretomes. The manual inspection of proteins represented with each ontological term assisted the identification of factors of the protein dataset belonging to secreted protease (SP), ECM components (E), soluble signaling factors (SF), and proteins involved in anabolic or catabolic processes (M).

During the manual inspection, we could further analyze the components of each ontological class to better understand whether proteins were associated with SASP functions. According to recent findings, SASP factors can be divided into soluble signaling factors (i.e., interleukins, chemokines, and growth factors), secreted proteases that remodel the extracellular matrix (ECM), ECM components, and proteins involved in anabolic and catabolic processes [16, 22, 23]. Indeed, in naïve secretomes, we found proteins belonging to all four SASP categories, while in primed secretomes we identified only components of ECM (Table 1).

Since annotations in GO databases are often redundant, functional annotation charts of GO analysis show relevant annotations repeatedly. This circumstance complicated identifying the most significant ontological class or classes in a given experimental condition.

**Figure 3:**
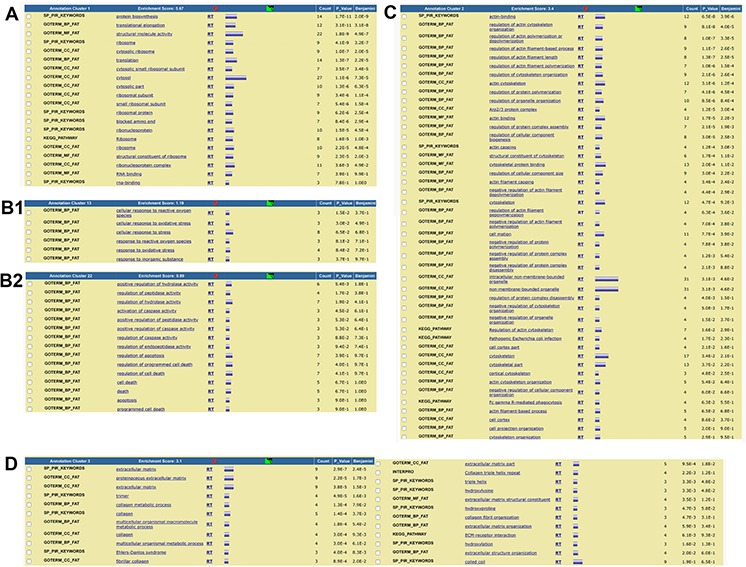
Summary of functional annotation clustering analysis (DAVID) The clustering of ontological terms found in the naïve secretome dataset and related to **A.** anabolic and catabolic processes, **B1.** senescence and **B2.** apoptosis, and **C.** ECM formation and remodeling; **D.** The clustering of ontological terms found in the primed secretome dataset and related to ECM formation and remodeling.

Fortunately, bioinformatics from the Database for Annotation, Visualization and Integrated Discovery (DAVID) now provides a functional annotation tool (i.e., functional annotation clustering) to reduce redundancy. This tool displays similar annotations together in order to determine GO terms that are either over- or under-represented in a given dataset. Functional annotation clustering identified 44 clusters in naïve secretomes and 29 in primed ones (Supplementary File S4). In this analysis, each cluster receives a group enrichment score, and top-ranked clusters are most likely to have consistently lower *p* values for their annotation members and thus indicate which ontology or ontologies could factor into specific conditions. In naïve secretomes, we found several clusters possibly associated with catabolic or anabolic processes, cytoskeleton and ECM remodeling, senescence, apoptosis, and autophagic activities (Figure 3; Supplementary File S4). In primed secretomes, however, we found no clusters associated with senescence or apoptosis. Clusters associated with ECM and cytoskeletons contain members previously described as regulators of cancer progression and metastasis (Figure 3; Supplementary File S4). Globally, the results of this analysis agreed with those found using PANTHER.

**Figure 4:**
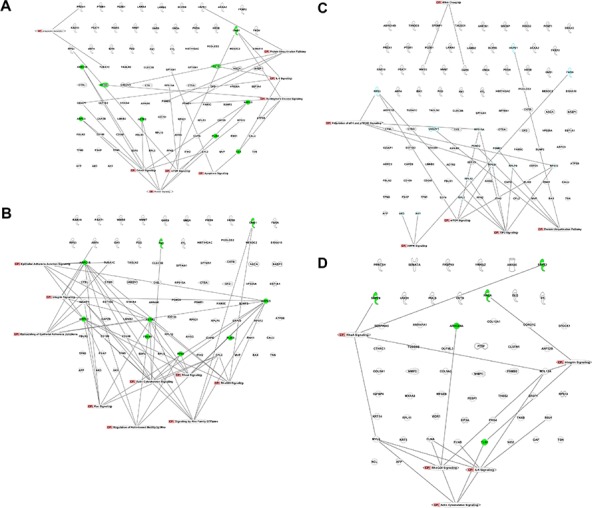
Canonical pathways identified with IPA Pathways in the naïve secretome dataset associated with **A.** senescence and apoptosis, **B.** ECM formation and remodeling, and **C.** anabolic and catabolic processes; in green are proteins with a clear role in senescence and/or apoptosis according to data from the literature; **D.** Pathways in the primed secretome dataset associated with ECM formation and remodeling; in green are proteins with a clear role in cancer growth and metastasis formation.

Since ontology analysis cannot rank individual proteins based on their significance in a process of interest, we used Ingenuity Pathway Analysis (IPA) for a preliminary evaluation of protein networks in our datasets. We focused on pathways possibly related to ontological classes identified by using the PANTHER database and DAVID. In naïve secretomes, we found several proteins belonging to networks potentially associated with senescence, apoptosis, and autophagy, including ARPC1B, ARPC3, ARPC5, ACTR2, BAX, CTSD, CSTS, and GNB1 (Figure 4). In primed secretomes, however, these networks were absent. We found pathways of epithelial adherens junction signaling, RhoGDI signaling, and Rac signaling, among others, that can relate to ECM formation and remodeling in both naïve and primed secretomes. In naïve secretomes, several of these proteins are involved in senescence-associated ECM remodeling. On the contrary, in primed samples we found some ECM-related proteins that contribute to cancer growth and progression (Figure 4). Lastly, in naïve secretomes, we identified several networks associated with catabolic or anabolic functions with numerous proteins described to factor into senescence, apoptosis, and autophagy (Figure 4).

## DISCUSSION

The principal result of our findings is that the evaluated naïve senescent MSC secretomes lack any stimulus for cancer cell growth. At the same time, the senescent secretomes obtained with different stressors induced senescence or apoptosis, if not both. The induction of apoptosis was associated with foci of persistent DNA damage in the cancer cell nuclei, suggesting that such damage is a prerequisite for the phenomenon. On the contrary, the accumulation of heavy DNA damage may be unnecessary for triggering senescence, possibly indicating that even if cells could repair DNA, such repair showed no fidelity and thus triggered senescence. In particular, ARH-77 cells mutated the p53 protein [24], implying that molecular controls of growth arrest, senescence, and apoptosis may occur through p53-independent pathways [1, 25, 26].

The great majority of research on the role of SASP in cancer has focused on the fact that cancer cells may misuse the secretome of senescent cells. For instance, SASPs from senescent fibroblasts have been involved with fostering cancer growth [3, 7–9, 15, 16]. Nevertheless, other findings have revealed that senescent fibroblasts may trigger the growth arrest and apoptosis of cancer cells *in vitro*, as we detected with naïve MSC secretomes in the present study [27]. These contradictory findings can be reconciled by considering that pro-growth effects observed were obtained with senescent cells co-cultivated in the presence of cancer cells. In other experiments, senescent cells were injected *in vivo* and reached tumor stroma. In both cases, senescent cells were primed with cancer cells, which may have forced senescent cells to produce cytokines and factors for survival and growth.

We sought to confirm this hypothesis by priming senescent cells with cancer and evaluating the effect of their secretomes upon cancer growth. Priming senescent cells with cancer cells modified their secretome composition in a way that rendered senescent MSCs ineffective in blocking cancer growth.

Our data concerning the importance of preliminary interaction between cancer cells and senescent cells are further supported by the pioneering studies of Nefedova et al. [28]. Their research has shown that CM from mononuclear bone marrow cells alone was insufficient for protecting myeloma cells from drug-induced apoptosis, whereas soluble factors produced during co-culture stimulated the proliferation of the cells. Moreover, depending on whether the priming procedure involved direct cell–cell contact, senescent cells produced different effects on myeloma cells.

Our results seem to indicate how cancer cells can misuse senescent secretome: they have to induce a change in protein production by interacting with senescent cells. Indeed, this interaction profoundly modified the composition of secretomes. Globally, priming induced the production of 55 proteins and repressed the expression (or secretion) of 102 others. Repressed ones belonged to networks associated with senescence, apoptosis, and catabolic or anabolic processes, while expressed ones belonged to ECM networks involved in cancer growth and the promotion of metastasis. Importantly, instead of promoting the production of factors boosting cancer growth, priming appeared to preclude the release of anti-cancer factors that could induce senescence or apoptosis.

To the best of our knowledge, we have thus shown for the first time that naïve senescent MSCs may have different effects on tumor progression compared with cancer-primed MSCs.

### Further studies and perspectives

Our findings raise some other issues. For one, key factors that may promote the abrogation of anti-tumor activity of naïve secretomes remain unidentified, as do factors released by cancer cells that affect senescent MSC functions. Moreover, it is unclear whether direct contact among cancer cells and senescent MSCs is a pre-requisite for priming. Two other questions are whether naïve senescent MSC secretomes arrest the growth of other cancer types and whether priming influences other senescent cell types such as fibroblasts. By contrast, it is possible that senescent fibroblasts are more prone to foster cancer growth than MSCs? Lastly, the role of secretome RNA components has yet to be pinpointed and its effects explained.

## CONCLUSIONS

In sum, the senescent secretome of naïve adipose-derived MSCs promoted senescence or apoptosis in myeloma cells. Both acute and chronic senescent secretomes demonstrated an ability to impair cancer cell growth, suggesting that such is a specific, conserved, core activity of naïve senescent MSCs. Cancer priming of senescent MSCs also abolished their anti-tumor activity. Lastly, that senescent MSC secretome may engage in pro-tumorigenic activity if these cells come into contact with cancer cells could constitute a critical threat to the safety of MSC transplants for therapeutic purposes in patients with cancer.

## MATERIALS AND METHODS

### MSC cultures

Lipoaspirates were obtained from healthy donors undergoing plastic surgery after they provided their informed consent. The dispersion of adipose tissue was achieved via collagenase digestion, after which the lipid-filled adipocytes' ability to float caused them to separate from the stromal vascular fraction by way of centrifugation. Stromal pellets were washed with phosphate-buffered saline (PBS) and further purified on a density gradient (Histopaque, GE Healthcare, UK). Mononuclear cells fractions were collected and cultivated in in Dulbecco's modified Eagle's medium containing 10% fetal bovine serum. These cells (passage 0) were further amplified to conduct experiments at passages 2 and 10 (i.e., replicative senescence).

### Acute senescence induction

We previously demonstrated that H_2_O_2_ or doxorubicin treatments can induce MSC senescence [29]. Following methods used in that study, adipose MSC cultures (passage 2) were treated with 300 μM H_2_O_2_ for 30 min, after which the medium was removed and the complete medium added. After 24 hours, the cells were used to prepare the CM.

To induce double-strand DNA breaks, MSCs were incubated for 16 hours with 1 μM doxorubicin. Following treatment, the medium was removed and the complete medium added. After 24 hours, the cells were also used to prepare the CM.

### Preparation of CM from naïve and primed senescent MSCs

To harvest naïve senescent MSC secretomes, 80% confluent MSC cultures were washed extensively with PBS and transferred to a chemically defined, serum-free culture medium for overnight incubation. Afterward, CM containing the MSC secretion were collected and used to supplement the *in vitro* cultivation of cancer cells.

We collected secretomes from acutely senescent MSCs (passage 2 cells treated with H_2_O_2_ or doxorubicin) and replicatively senescent MSCs (passage 10 cells). Using *in situ* senescence-associated β-galactosidase assay, we confirmed that our treatments induced senescence in MSC cultures. For a control culture, we collected CM from naïve healthy MSCs (passage 2).

To prepare CM from cancer-primed cells, we co-cultivated in a 1:1 ratio acutely or chronically senescent MSCs in direct contact with ARH-77 cells for 24 hours. Thereafter, ARH-77 cells were discarded and the senescent MSC cultures purified with PBS. The ARH-77-primed senescent MSCs were subsequently transferred to a chemically defined medium for overnight incubation, as also done with naïve senescent cells to produce CM. For a control culture, we collected CM from cancer-primed healthy MSCs (passage 2).

### Cancer cell lines and treatment with CM

ARH-77 multiple myeloma were grown exponentially in RPMI-1640 medium of 10% serum. To evaluate the effect of CM on the *in vitro* behavior of cancer cells, exponentially growing cultures were incubated in their respective media supplemented with 50% CM obtained from both naïve and primed senescent MSCs. After cancer cells stayed in these conditions for 48 hours, we performed the biological assays described below.

### Cell cycle analysis

For each assay, cells were collected, fixed in 70% ethanol, washed with PBS, and dissolved in a hypotonic buffer containing propidium iodide. Samples were collected using a flow cytometer (Muse EasyCyte, Merck Millipore, Germany) and analyzed with EasyCyte software according to the standard procedure.

### Annexin V assay

Apoptotic cells were detected by using a fluorescein-conjugated annexin V kit on a Muse EasyCyte flow cytometer following the manufacturer's instructions.

### *In situ* senescence-associated β-galactosidase assay

The percentage of senescent cells was calculated by the number of blue, β-galactosidase-positive cells among at least 500 cells in different microscopic fields, as both previously reported [30] and detailed in Supplementary File S5.

### Immunocytochemistry for detecting gamma-H2AX and Ki-67

Phosphorylated H2AX (gamma-H2AX) and Ki-67 were detected according to manufacturers' protocols. Cells were stained with Hoechst 33342 and observed using a fluorescence microscope (Leica Italia, Italy). The percentage of Ki-67-positive cells was calculated by counting at least 500 cells among the different microscopic fields, and the degree of H2AX phosphorylation was evaluated by counting the number of gamma-H2AX immunofluorescent foci per cell. Each foci number was determined for 200 cells (Supplementary File S5).

### CM preparation for LC-MS/MS analysis

Without disturbing the attached cells, 5 mL of naïve or ARH-77-primed MSC secretomes were collected from culture dishes and culture debris removed by centrifugation at 10,000 *g*. Supernatants were used for protein pooling with resin (StrataClean, Agilent Technology, CA, USA) using dried beads mixed with 1× Laemmli gel loading buffer and run on a gradient gel 4–15% SDS-PAGE (Criterion TGX Stain-Free Precast Gels, Bio-Rad, CA, USA). Following electrophoresis at 100 V, the gels were stained with Coomassie Brilliant Blue and gel lanes of interest excised for in-gel digestion, as previously described [31, 32].

After digestion, peptides were eluted from the gel matrix by immersing the reaction tube in an ultrasonic bath for 5 min with a sequential elution of 0.4% formic acid in 3% ACN, 0.4% formic acid in 50% ACN, and 100% ACN. The supernatant containing the peptides was centrifuged, transferred to low binding tubes, and desalted by using pipette tips (ZipTip C18, Merck Millipore, Germany). Following that, extracted peptides were dried and stored at −80°C until LC-MS/MS analysis was performed. A more detailed protocol of CM preparation appears in Supplementary File S5.

### LC-MS/MS analysis

Tandem mass spectrometric analysis was carried out using AB SCIEX TripleTOF 5600+ instrument (AB SCIEX, Redwood City, CA, USA) coupled to Eksigent expert nano-LC 400 system (AB SCIEX). MS and MS/MS data was acquired using Analyst^®^ TF v.1.6 (AB SCIEX).

Mass spectrometry data was analyzed by using ProteinPilot 4.5 Beta (AB SCIEX) for the peptide identifications. Detailed protocol in Supplementary File S5. The mass spectrometry proteomics data have been deposited to the ProteomeXchange Consortium [33] via the PRIDE partner repository with the dataset identifier PXD003018.

### GO and network analyses

Proteins expressed in secretomes were analyzed with PANTHER (http://www.pantherdb.org), DAVID, http://david.abcc.ncifcrf.gov), and IPA (http://www.ingenuity.com/product/ipa).

Using PANTHER, protein classification was performed according to three ontological terms: biological processes, molecular functions, and molecular classes. For PANTHER analysis, we used statistics overrepresentation (i.e., the default setting) to compare classifications of multiple clusters of lists with a reference list to statistically identify the over- or under-representation of PANTHER ontologies. Significance was set to a *p* value of 0.05.

With DAVID, we used functional annotation clustering to identify proteins in the most important biological functional groups. In this database, a significant enrichment score for any group indicates that its proteins are involved in a cluster with a possibly significant function in a given dataset. The parameters for clustering referred to medium classification stringency (EASE enrichment threshold: 1.0). Clusters were ranked according a group enrichment score calculated from the geometric mean of a member's *p* values in a corresponding annotation cluster.

Differentially expressed proteins were imported into IPA to identify canonical pathways present exclusively in either naïve or primed secretomes. Fischer's exact test was used to calculate a *p* value that would determine the probability that the association between genes in the dataset and canonical pathway could be explained by chance alone. Significance was set to 0.05.

### Statistical analysis

Statistical significance was determined by using one-way analysis of variance followed by Bonferroni's tests. Mixed-model variance analysis was used for data with continuous outcomes. All data were analyzed with a statistics software package (GraphPad Prism version 5.01, GraphPad, CA, USA).

## SUPPLEMENTARY FILES











## References

[1] Campisi J, d'Adda di Fagagna F (2007). Cellular senescence: when bad things happen to good cells. Nat Rev Mol Cell Biol.

[2] van Deursen JM (2014). The role of senescent cells in ageing. Nature.

[3] Coppe JP, Patil CK, Rodier F, Sun Y, Munoz DP, Goldstein J, Nelson PS, Desprez PY, Campisi J (2008). Senescence-associated secretory phenotypes reveal cell-nonautonomous functions of oncogenic RAS and the p53 tumor suppressor. PLoS biology.

[4] Fumagalli M, d'Adda di Fagagna F (2009). SASPense and DDRama in cancer and ageing. Nature cell biology.

[5] Kuilman T, Peeper DS (2009). Senescence-messaging secretome: SMS-ing cellular stress. Nature reviews Cancer.

[6] Velarde MC, Demaria M, Campisi J (2013). Senescent cells and their secretory phenotype as targets for cancer therapy. Interdisciplinary topics in gerontology.

[7] Coppe JP, Kauser K, Campisi J, Beausejour CM (2006). Secretion of vascular endothelial growth factor by primary human fibroblasts at senescence. The Journal of biological chemistry.

[8] Krtolica A, Parrinello S, Lockett S, Desprez PY, Campisi J (2001). Senescent fibroblasts promote epithelial cell growth and tumorigenesis: a link between cancer and aging. Proceedings of the National Academy of Sciences of the United States of America.

[9] Liu D, Hornsby PJ (2007). Senescent human fibroblasts increase the early growth of xenograft tumors via matrix metalloproteinase secretion. Cancer research.

[10] Galderisi U, Giordano A (2014). The gap between the physiological and therapeutic roles of mesenchymal stem cells. Medicinal research reviews.

[11] Giordano A, Galderisi U, Marino IR (2007). From the laboratory bench to the patient's bedside: an update on clinical trials with mesenchymal stem cells. Journal of cellular physiology.

[12] Ranganath SH, Levy O, Inamdar MS, Karp JM (2012). Harnessing the mesenchymal stem cell secretome for the treatment of cardiovascular disease. Cell stem cell.

[13] Severino V, Alessio N, Farina A, Sandomenico A, Cipollaro M, Peluso G, Galderisi U, Chambery A (2013). Insulin-like growth factor binding proteins 4 and 7 released by senescent cells promote premature senescence in mesenchymal stem cells. Cell death & disease.

[14] Galderisi U, Giordano A, Paggi MG (2010). The bad and the good of mesenchymal stem cells in cancer: Boosters of tumor growth and vehicles for targeted delivery of anticancer agents. World journal of stem cells.

[15] Bavik C, Coleman I, Dean JP, Knudsen B, Plymate S, Nelson PS (2006). The gene expression program of prostate fibroblast senescence modulates neoplastic epithelial cell proliferation through paracrine mechanisms. Cancer research.

[16] Coppe JP, Desprez PY, Krtolica A, Campisi J (2010). The senescence-associated secretory phenotype: the dark side of tumor suppression. Annual review of pathology.

[17] Mastri M, Lin H, Lee T (2014). Enhancing the efficacy of mesenchymal stem cell therapy. World journal of stem cells.

[18] Waterman RS, Tomchuck SL, Henkle SL, Betancourt AM (2010). A new mesenchymal stem cell (MSC) paradigm: polarization into a pro-inflammatory MSC1 or an Immunosuppressive MSC2 phenotype. PloS one.

[19] Yan H, Wu M, Yuan Y, Wang ZZ, Jiang H, Chen T (2014). Priming of Toll-like receptor 4 pathway in mesenchymal stem cells increases expression of B cell activating factor. Biochemical and biophysical research communications.

[20] Raposo G, Stoorvogel W (2013). Extracellular vesicles: exosomes, microvesicles, and friends. J Cell Biol.

[21] Villarreal L, Mendez O, Salvans C, Gregori J, Baselga J, Villanueva J (2013). Unconventional secretion is a major contributor of cancer cell line secretomes. Mol Cell Proteomics.

[22] Quijano C, Cao L, Fergusson MM, Romero H, Liu J, Gutkind S, Rovira II, Mohney RP, Karoly ED, Finkel T (2012). Oncogene-induced senescence results in marked metabolic and bioenergetic alterations. Cell Cycle.

[23] Salama R, Sadaie M, Hoare M, Narita M (2014). Cellular senescence and its effector programs. Genes Dev.

[24] Drewinko B, Mars W, Minowada J, Burk KH, Trujillo JM (1984). ARH-77, an established human IgG-producing myeloma cell line. I. Morphology, B-cell phenotypic marker profile, and expression of Epstein-Barr virus. Cancer.

[25] Amaral JD, Xavier JM, Steer CJ, Rodrigues CM (2010). The role of p53 in apoptosis. Discovery medicine.

[26] Liebermann DA, Hoffman B, Steinman RA (1995). Molecular controls of growth arrest and apoptosis: p53-dependent and independent pathways. Oncogene.

[27] Vjetrovic J, Shankaranarayanan P, Mendoza-Parra MA, Gronemeyer H (2014). Senescence-secreted factors activate Myc and sensitize pretransformed cells to TRAIL-induced apoptosis. Aging cell.

[28] Nefedova Y, Landowski TH, Dalton WS (2003). Bone marrow stromal-derived soluble factors and direct cell contact contribute to de novo drug resistance of myeloma cells by distinct mechanisms. Leukemia.

[29] Zanichelli F, Capasso S, Di Bernardo G, Cipollaro M, Pagnotta E, Carteni M, Casale F, Iori R, Giordano A, Galderisi U (2012). Low concentrations of isothiocyanates protect mesenchymal stem cells from oxidative injuries, while high concentrations exacerbate DNA damage. Apoptosis: an international journal on programmed cell death.

[30] Alessio N, Bohn W, Rauchberger V, Rizzolio F, Cipollaro M, Rosemann M, Irmler M, Beckers J, Giordano A, Galderisi U (2013). Silencing of RB1 but not of RB2/P130 induces cellular senescence and impairs the differentiation potential of human mesenchymal stem cells. Cellular and molecular life sciences: CMLS.

[31] Shevchenko A, Tomas H, Havlis J, Olsen JV, Mann M (2006). In-gel digestion for mass spectrometric characterization of proteins and proteomes. Nature protocols.

[32] Bonn F, Bartel J, Büttner K, Hecker M, Otto A, Becher D (2014). Picking vanished proteins from the void: how to collect and ship/share extremely dilute proteins in a reproducible and highly efficient manner. Analytical Chemistry.

[33] Vizcaíno JA, Deutsch EW, Wang R, Csordas A, Reisinger F, Ríos D, Dianes JA, Sun Z, Farrah T, Bandeira N, Binz PA, Xenarios I, Eisenacher M, Mayer G, Gatto L, Campos A, Chalkley RJ, Kraus HJ, Albar JP, Martinez-Bartolomé S, Apweiler R, Omenn GS, Martens L, Jones AR, Hermjakob H (2014). ProteomeXchange provides globally co-ordinated proteomics data submission and dissemination. Nature Biotechnology.

