# Supplementary material for: Myeloma cells can corrupt senescent mesenchymal stromal cells and impair their anti-tumor activity
Source: Oncotarget. 2015 Oct 16;6(37):39482–92. doi: 10.18632/oncotarget.5430 (PMC4741840; doi:10.18632/oncotarget.5430)
Supplement: Supplementary file 1 [file oncotarget-06-39482-s001.pdf]

SUPPLEMENTARY FILES

Supplementary File S1: List of proteins that are present in naïve secretomes obtained from doxorubicin- or H_2O_2-treated and replicatively senescent MSCs.

Supplementary File S2: List of proteins present in primed secretomes obtained from doxorubicin- or H_2O_2-treated and replicatively senescent MSCs.

Supplementary File S3: Results of GO slim analysis performed with PANTHER listed in ontological terms classified into biological processes, molecular functions, and molecular classes that were overrepresented in naïve and primed secretomes.

Supplementary File S4: List of enriched clusters of Gene Ontology terms identified in naïve and primed secretomes with DAVID's functional annotation clustering tool.

Supplementary File S5: Detailed protocol of some methods.

Senescence-associated β-galactosidase staining

The Senescence β-Galactosidase Staining is designed to detect β-galactosidase activity at pH 6, a known characteristic of senescent cells not found in prese- nescent, quiescent or immortal cells (Dimri, Lee et al. 1995).

Cultured cells were washed twice with PBS and then fixed for 10 minutes with 2% formaldehyde/0.2% glutaraldehyde at room temperature. Cells were then washed twice with PBS and 1 to 2 ml of senescence-associated β-galactosidase staining solution per 35 mm dish was added. Cells were incubated at 37°C for several hours until blue staining was clearly detectable under a light microscope. The percentage of senescent cells was calculated by the number of β-galactosidase-positive cells out of at least 500 cells in different microscope fields. (Just before using the senescence-associated β-galactosidase staining solution, we added 20 ml of citric acid/sodium phosphate (pH 6) in a volume of 100 ml of 150 mM NaCl, 2 mM MgCl2, 5 mM potassium ferricyanide, 5 mM potassium ferrocyanide and 1 mg/ml X-Gal.)

Immunocytochemistry for gamma H2AX and Ki67

The histone H2AX is rapidly phosphorylated following DNA damage by ATM protein that is a component of DNA repair system. The phosphorylated H2AX (gamma-H2AX) is involved in the recruitment and/or retention of DNA repair and checkpoint proteins. Gamma-H2AX foci are marker of damaged DNA that is undergoing the repair process, and the persistence of these foci several hours (or days) following genotoxic stress is a sign of the presence of unrepaired DNA in cells' nuclei.

Rabbit monoclonal anti-phospho histone H2A.X (pH2A.X), (Cell Signaling, Denvers, MA, USA) antibodies were used for the detection of pH2A.X according to the manufacturer's protocol. Nuclei were counterstained with Hoechst 33342 and cells were observed through a fluorescence microscope (Leica, Milan, Italy). The degree of H2AX phosphorylation was evaluated by counting the number of gamma-H2AX immunofluorescent foci per cell. Foci number was determined for 200 cells.

We carried out Ki67 immunostaining to detect the percentage of cycling (G1; S; G2/M) and resting cells (G0) that are Ki67 positive and negative, respectively.(Jansen, Hupperets et al. 1998).

Mouse monoclonal anti-Ki67 Cell Signaling, Denvers, MA, USA antibodies were used for the detection of Ki67 according to the manufacturer's protocol. Nuclei were counterstained with Hoechst 33342 and cells were observed through a fluorescence microscope (Leica, Milan,

Italy). The percentage of Ki67-positive cells was calculated by counting at least 500 cells in different microscope fields.

CM preparation for LC-MS/MS analysis

Binding of proteins on StrataClean beads

StrataClean resin is a phenol-free technique for DNA purification (Agilent Technology, CA, USA). The solid phase silica-based resin contains hydroxyl groups that react with proteins in much the same manner as the hydroxyl group of phenol. It is normally used to separate DNA from all restriction enzymes and some modifying enzymes. Beads can also be used to concentrate isolate proteins that bind silica resin.

Five mL of naïve or ARH77-primed MSC secretomes were collected from culture dishes without disturbing the attached cells, then culture debris were removed by centrifugation 10000 g at 4°C. Supernatants of secretomes were either used for the StartaClean beads protein pooling or stored at–80°C for further use. 20 µL of StrataClean beads (Agilent, USA) was added into 5 mL of each secretome sample, then incubated at 4°C overnight with gentle shaking or overhead rotation. The beads were precipitated by centrifugation at 8000 g at 4°C for 15 min. Then supernatants were removed, the protein-loaded beads were washed two times with 500 µL of TE (50 mM Tris, 10 mM EDTA, pH 8), transferred to a low binding microfuge tube and the beads were evaporated to dryness with a vacuum centrifuge.

Protein Elution with SDS-PAGE

Twenty µL of 1x Laemmli gel loading buffer supplemented with 20 mM DTT was added to dried affinity beads. The mixture slurry was boiled for 5 min, chilled on ice and total mixture loaded on a gradient gel 4–15% SDS-PAGE (Criterion TGX Stain Free Precast Gels, BIO-RAD, US). Electrophoresis was carried out at 100 V. The gel was pre-scanned (ChemiDocTM MP, BIO-RAD, US) then fixed with 10% (v/v) acetic acid in 40% (v/v) ethanol for 60 min and stained with colloidal Coomassie blue. After staining, the gel was rinsed twice with ddH2O to remove excessive Coomassie stain. The gel lanes of interest were excised. For each loading well, six to ten bands were excised and destained. In-gel digestion was carried out as described previously (Shevchenko, Tomas et al. 2006). After digestion, the peptides were eluted from the gel matrix by immersion of the reaction tube in an ultrasonic bath for 5 min with sequentially elution of 0.4% formic acid in 3% ACN, 0.4% formic acid in 50% ACN and 100% ACN. The supernatant containing the peptides was centrifuged, transferred to low binding tubes, desalted with ZipTip C18 (Millipore, Merck). Then, the extracted peptides were dried and stored at –80°C until LC-MS/MS analysis.

LC-MS/MS analysis

Tandem mass spectrometric analysis was carried out using AB SCIEX TripleTOF 5600+ instrument (AB SCIEX, Redwood City, CA, USA) coupled to Eksigent expert nano-LC 400 system (AB SCIEX). Trap and elute mode was used to separate peptide mixture by LC system equipped with a trap column (180 µm × 20 mm column, 300 Å, nanoACQUITY UPLC® 2G-VM Trap 5µm Symetry® C18, Waters, UK) and a separation column (75 µm, x 150mm column, nanoACQUITY UPLC® 1.8 µm 120 Å HSS T3, C18, Waters, UK). Two µL of peptide samples were loaded onto the LC system with 10 µL loop valume in solvent A (0.1% formic acid (v/v) in H2O) for 10 min at a constant flow rate of 1 µL/min with trapping. The peptide elution carried out using a linear gradient of 4–96% solvent B (0.1% formic acid in 100% ACN) for 130 minutes at a constant flow rate of 250 nL/min. The gradient program was used as follows: the system was preconditioned with 96% solvent A (0.1% formic acid (v/v) in H2O) for 10 min. Following, solvent B (0.1% formic acid in 100% ACN) was increase from 4% to 50% with the duration of 95 min. Then, solvent B increased to 96% within 6 min; maintained for 7 min at 96% B / 4% A; then cleaning for the next run was established. Elute was delivered into the mass spectrometer with a NanoSpray III source using a 10 µm ID nanospray emitter tip (New Objective, Woburn, MA). Electrospray ionization of floating voltage was maintained at 2400 V.

MS and MS/MS data was acquired using Analyst® TF v.1.6 (AB SCIEX). After a survey scans at a resolution of >35K in the m/z range of 350 to 1250 which exceeds 70 cps, high sensitivity on MS/MS mode with resolution in the m/z range of 100 to 1800 on both precursor and fragment of the 20 most abundant precursor ions were selected for fragmentation. A rolling Collision-induced dissociation (CID) fragmentation was performed for 25 ms with normalized collision energy of 10, declustering potential was set to 100 V and the fragment ions were recorded. Single and unassigned charge state precursor ions were not selected for MS/MS analysis. Mass tolerance was set to ±50 mDa. Mass spectrometer recalibration was performed at the start of each batch and repeated every third sample by using 25 fmol/µL β-galactosidase digest standard.

Mass spectrometry data analysis

Mass spectrometry data was analyzed by using ProteinPilot 4.5 Beta (AB SCIEX) for the peptide identifications. Following the extraction of all files, *.wiff files were used to carry out the peak list generation and database searches. In house human protein database was generated from UniProt Proteomes reference database of Homo sapiens (UP000005640, downloaded January 16, 2015). The parameters were used for database searches

include trypsin as a protease with the allowance of one missed cleavage, and oxidation of methionine. Precursor ion mass error window of 10 ppm and fragment ion mass error window of 0.1 Da were allowed. The raw data obtained were searched against decoy database to calculate 1% false discovery (FDR). Proteins were only considered as identified if at least two unique peptides of which matching to a protein.

REFERENCES

Dimri, G. P., X. Lee, G. Basile, M. Acosta, G. Scott, C. Roskelley, E. E. Medrano, M. Linskens, I. Rubelj, O. Pereira-Smith, et al. "A biomarker that identifies senescent human cells in culture and in aging skin *in vivo*." Proc Natl Acad Sci U S A. 1995; 92:9363–9367.

Jansen, R. L., P. S. Hupperets, J. W. Arends, S. R. Joosten-Achjanie, A. Volovics, H. C. Schouten, H. F. Hillen. "MIB-1 labelling index is an independent prognostic marker in primary breast cancer." Br J Cancer. 1998; 78:460–465.

Shevchenko, A., H. Tomas, J. Havlis, J. V. Olsen, M. Mann. "In-gel digestion for mass spectrometric characterization of proteins and proteomes." Nat Protoc. 2006; 1:2856–2860.
