# Supplementary material for: Myeloma cells can corrupt senescent mesenchymal stromal cells and impair their anti-tumor activity
Source: Oncotarget. 2015 Oct 16;6(37):39482–92. doi: 10.18632/oncotarget.5430 (PMC4741840; doi:10.18632/oncotarget.5430)
Supplement: Supplementary file 4 [file oncotarget-06-39482-s004.pdf]

Now includes comprehensive GO annotations directly imported from the GO database

Analysis Summary: Please report in publication

Analysis Type: PANTHER Overrepresentation Test (release 20150430)

Annotation Version and Release Date: PANTHER version 10.0 Released 2015-05-15

Analyzed List: **Naïve secretomes** Change

Reference List: Homo sapiens (all genes in database) Change

Annotation Data Set: PANTHER GO-Slim Biological Process

☑ Use the Bonferroni correction for multiple testing

Results

	Reference list	Client Text Box Input
Mapped IDs:	20814	101
Unmapped IDs:	0	0

Export results View: -- Please select a chart to display --

Displaying only results with P<0.05; click here to display all results

	Homo sapiens (REF)	Client Text Box Input				
PANTHER GO-Slim Biological Process	#	#	expected	Fold Enrichment	+/-	P value
translation	435	12	2.11	> 5	+	3.26E-04
proteolysis	719	12	3.49	3.44	+	4.38E-02
protein metabolic process	2692	36	13.06	2.76	+	1.18E-06
metabolic process	8247	62	40.02	1.55	+	1.81E-03
primary metabolic process	6825	51	33.12	1.54	+	3.83E-02
Unclassified	8629	13	41.87	.31	-	0.00E00

© Copyright 2015 Paul Thomas All Rights Reserved.

Now includes comprehensive GO annotations directly imported from the GO database

Analysis Summary: Please report in publication

Analysis Type: PANTHER Overrepresentation Test (release 20150430)

Annotation Version and Release Date: PANTHER version 10.0 Released 2015-05-15

Analyzed List: Naïve secretomes (Change)

Reference List: Homo sapiens (all genes in database) (Change)

Annotation Data Set: PANTHER GO-Slim Molecular Function

☑ Use the Bonferroni correction for multiple testing

Results

	Reference list	Client Text Box Input
Mapped IDs:	20814	101
Unmapped IDs:	0	0

Export results View: -- Please select a chart to display --

Displaying only results with P<0.05; click here to display all results

	Homo sapiens (REF)	Client Text Box Input				
PANTHER GO-Slim Molecular Function	#	#	expected	Fold Enrichment	+/-	P value
actin binding	176	7	.85	> 5	+	4.49E-03
cytoskeletal protein binding	251	7	1.22	> 5	+	4.01E-02
calcium ion binding	449	11	2.18	> 5	+	2.12E-03
structural molecule activity	1034	21	5.02	4.19	+	3.79E-06
structural constituent of cytoskeleton	663	12	3.22	3.73	+	1.58E-02
Unclassified	10020	21	48.62	.43	-	0.00E00

© Copyright 2015 Paul Thomas All Rights Reserved.

Now includes comprehensive GO annotations directly imported from the GO database

Analysis Summary: Please report in publication

Analysis Type: PANTHER Overrepresentation Test (release 20150430)

Annotation Version and Release Date: PANTHER version 10.0 Released 2015-05-15

Analyzed List: **Naïve secretomes** Change

Reference List: Homo sapiens (all genes in database) Change

Annotation Data Set: PANTHER Protein Class

Use the Bonferroni correction for multiple testing

Results

	Reference list	Client Text Box Input
Mapped IDs:	20814	101
Unmapped IDs:	0	0

Export results View: -- Please select a chart to display --

Displaying only results with P<0.05; click here to display all results

	Homo sapiens (REF)	Client Text Box Input				
PANTHER Protein Class	#	#	expected	Fold Enrichment	+/-	P value
ribosomal protein	225	8	1.09	> 5	+	3.13E-03
actin family cytoskeletal protein	395	12	1.92	> 5	+	1.10E-04
cytoskeletal protein	806	14	3.91	3.58	+	7.41E-03
Unclassified	9675	20	46.95	.43	-	0.00E00

© Copyright 2015 Paul Thomas All Rights Reserved.

Now includes comprehensive GO annotations directly imported from the GO database

Analysis Summary: Please report in publication

Analysis Type: PANTHER Overrepresentation Test (release 20150430)

Annotation Version and Release Date: PANTHER version 10.0 Released 2015-05-15

Analyzed List: Primed secretomes — Change

Reference List: Homo sapiens (all genes in database) — Change

Annotation Data Set: PANTHER GO-Slim Biological Process

☑ Use the Bonferroni correction for multiple testing

Results

	Reference list	Client Text Box Input
Mapped IDs:	20814	55
Unmapped IDs:	0	0

Export results   View: -- Please select a chart to display --

Displaying only results with P<0.05; click here to display all results

	Homo sapiens (REF)	Client Text Box Input				
PANTHER GO-Slim Biological Process	#	#	expected	Fold Enrichment	+/-	P value
cellular component morphogenesis	478	9	1.26	> 5	+	9.60E-04
anatomical structure morphogenesis	596	9	1.57	> 5	+	5.51E-03
developmental process	2456	17	6.49	2.62	+	2.94E-02
Unclassified	8629	16	22.80	.70	-	0.00E00

© Copyright 2015 Paul Thomas All Rights Reserved.

Now includes comprehensive GO annotations directly imported from the GO database

Analysis Summary: Please report in publication

Analysis Type: PANTHER Overrepresentation Test (release 20150430)

Annotation Version and Release Date: PANTHER version 10.0 Released 2015-05-15

Analyzed List: **Primed secretomes** Change

Reference List: Homo sapiens (all genes in database) Change

Annotation Data Set: PANTHER GO-Slim Molecular Function

☑ Use the Bonferroni correction for multiple testing

Results

	Reference list	Client Text Box Input
Mapped IDs:	20814	55
Unmapped IDs:	0	0

Export results View: -- Please select a chart to display --

Displaying only results with P<0.05; click here to display all results

	Homo sapiens (REF)	Client Text Box Input				
PANTHER GO-Slim Molecular Function	#	#	expected	Fold Enrichment	+/-	P value
structural constituent of cytoskeleton	663	11	1.75	> 5	+	1.89E-04
structural molecule activity	1034	16	2.73	> 5	+	1.08E-06
protein binding	2819	18	7.45	2.42	+	3.73E-02
Unclassified	10020	20	26.48	.76	-	0.00E00

© Copyright 2015 Paul Thomas All Rights Reserved.

Now includes comprehensive GO annotations directly imported from the GO database

Analysis Summary: Please report in publication

Analysis Type: PANTHER Overrepresentation Test (release 20150430)

Annotation Version and Release Date: PANTHER version 10.0 Released 2015-05-15

Analyzed List: **Primed secretomes** Change

Reference List: Homo sapiens (all genes in database) Change

Annotation Data Set: PANTHER Protein Class

☑ Use the Bonferroni correction for multiple testing

Results

	Reference list	Client Text Box Input
Mapped IDs:	20814	55
Unmapped IDs:	0	0

Export results View: -- Please select a chart to display --

Displaying only results with P<0.05; click here to display all results

	Homo sapiens (REF)	Client Text Box Input				
PANTHER Protein Class	#	#	expected	Fold Enrichment	+/-	P value
actin family cytoskeletal protein	395	7	1.04	> 5	+	1.64E-02
cytoskeletal protein	806	12	2.13	> 5	+	2.13E-04
Unclassified	9675	16	25.57	.63	-	0.00E00

© Copyright 2015 Paul Thomas All Rights Reserved.
