# Supplementary material for: Myeloma cells can corrupt senescent mesenchymal stromal cells and impair their anti-tumor activity
Source: Oncotarget. 2015 Oct 16;6(37):39482–92. doi: 10.18632/oncotarget.5430 (PMC4741840; doi:10.18632/oncotarget.5430)
Supplement: Supplementary file 5 [file oncotarget-06-39482-s005.pdf]

# Functional Annotation Clustering

Help and Manual

**Current Gene List: Naive secretomes**
**Current Background: Homo sapiens**
**101 DAVID IDs**

**Options** **Classification Stringency** Medium

Rerun using options | Create Sublist

**44 Cluster(s)** Download File

| Annotation Cluster 1 | Enrichment Score: 5.67 | | Count | P_Value | Benjamini |
|---|---|---|---|---|---|
| SP_PIR_KEYWORDS | protein biosynthesis | RT | 14 | 1.7E-11 | 2.0E-9 |
| GOTERM_BP_FAT | translational elongation | RT | 12 | 3.1E-11 | 3.1E-8 |
| GOTERM_MF_FAT | structural molecule activity | RT | 22 | 1.8E-9 | 4.9E-7 |
| SP_PIR_KEYWORDS | ribosome | RT | 9 | 4.1E-9 | 3.2E-7 |
| GOTERM_CC_FAT | cytosolic ribosome | RT | 9 | 1.0E-7 | 2.0E-5 |
| GOTERM_BP_FAT | translation | RT | 14 | 1.3E-7 | 2.2E-5 |
| GOTERM_CC_FAT | cytosolic small ribosomal subunit | RT | 7 | 3.5E-7 | 3.4E-5 |
| GOTERM_CC_FAT | cytosol | RT | 27 | 1.1E-6 | 7.3E-5 |
| GOTERM_CC_FAT | cytosolic part | RT | 10 | 1.3E-6 | 6.3E-5 |
| GOTERM_CC_FAT | ribosomal subunit | RT | 9 | 3.4E-6 | 1.1E-4 |
| GOTERM_CC_FAT | small ribosomal subunit | RT | 7 | 5.4E-6 | 1.5E-4 |
| SP_PIR_KEYWORDS | ribosomal protein | RT | 9 | 6.2E-6 | 2.5E-4 |
| SP_PIR_KEYWORDS | blocked amino end | RT | 7 | 8.4E-6 | 2.9E-4 |
| SP_PIR_KEYWORDS | ribonucleoprotein | RT | 10 | 1.5E-5 | 4.5E-4 |
| KEGG_PATHWAY | Ribosome | RT | 8 | 1.6E-5 | 1.0E-3 |
| GOTERM_CC_FAT | ribosome | RT | 10 | 2.2E-5 | 4.8E-4 |
| GOTERM_MF_FAT | structural constituent of ribosome | RT | 9 | 2.3E-5 | 2.0E-3 |
| GOTERM_CC_FAT | ribonucleoprotein complex | RT | 11 | 3.6E-3 | 4.9E-2 |
| GOTERM_MF_FAT | RNA binding | RT | 7 | 3.9E-1 | 9.9E-1 |
| SP_PIR_KEYWORDS | rna-binding | RT | 3 | 7.8E-1 | 1.0E0 |

| Annotation Cluster 2 | Enrichment Score: 3.4 | | Count | P_Value | Benjamini |
|---|---|---|---|---|---|
| SP_PIR_KEYWORDS | actin-binding | RT | 12 | 6.5E-8 | 3.9E-6 |
| GOTERM_BP_FAT | regulation of actin cytoskeleton organization | RT | 9 | 8.1E-8 | 4.0E-5 |
| GOTERM_BP_FAT | regulation of actin polymerization or depolymerization | RT | 8 | 1.0E-7 | 3.3E-5 |
| GOTERM_BP_FAT | regulation of actin filament-based process | RT | 9 | 1.1E-7 | 2.6E-5 |
| GOTERM_BP_FAT | regulation of actin filament length | RT | 8 | 1.3E-7 | 2.5E-5 |
| GOTERM_BP_FAT | regulation of actin filament polymerization | RT | 7 | 1.0E-6 | 1.5E-4 |
| GOTERM_BP_FAT | regulation of cytoskeleton organization | RT | 9 | 2.1E-6 | 2.6E-4 |
| GOTERM_CC_FAT | actin cytoskeleton | RT | 12 | 3.1E-6 | 1.2E-4 |
| GOTERM_BP_FAT | regulation of protein polymerization | RT | 7 | 4.1E-6 | 4.5E-4 |
| GOTERM_BP_FAT | regulation of organelle organization | RT | 10 | 8.5E-6 | 8.4E-4 |
| GOTERM_CC_FAT | Arp2/3 protein complex | RT | 4 | 1.2E-5 | 3.0E-4 |
| GOTERM_MF_FAT | actin binding | RT | 12 | 1.7E-5 | 2.2E-3 |
| GOTERM_BP_FAT | regulation of protein complex assembly | RT | 7 | 2.1E-5 | 1.9E-3 |
| GOTERM_BP_FAT | regulation of cellular component biogenesis | RT | 8 | 3.0E-5 | 2.5E-3 |
| SP_PIR_KEYWORDS | actin capping | RT | 4 | 1.2E-4 | 3.0E-3 |
| GOTERM_MF_FAT | structural constituent of cytoskeleton | RT | 6 | 1.7E-4 | 1.1E-2 |
| GOTERM_MF_FAT | cytoskeletal protein binding | RT | 13 | 2.0E-4 | 1.1E-2 |
| GOTERM_BP_FAT | regulation of cellular component size | RT | 9 | 3.0E-4 | 2.2E-2 |
| GOTERM_BP_FAT | actin filament capping | RT | 4 | 3.4E-4 | 2.4E-2 |
| GOTERM_BP_FAT | negative regulation of actin filament depolymerization | RT | 4 | 4.4E-4 | 2.9E-2 |
| SP_PIR_KEYWORDS | cytoskeleton | RT | 12 | 4.7E-4 | 9.2E-3 |
| GOTERM_BP_FAT | regulation of actin filament | RT | 4 | 6.3E-4 | 3.6E-2 |

| Category | Term | RT | Count | P_Value | Benjamini |
|---|---|---|---|---|---|
| | depolymerization | | | | |
| GOTERM_BP_FAT | negative regulation of actin filament polymerization | RT | 4 | 7.0E-4 | 3.8E-2 |
| GOTERM_BP_FAT | cell motion | RT | 11 | 7.7E-4 | 3.9E-2 |
| GOTERM_BP_FAT | negative regulation of protein polymerization | RT | 4 | 7.8E-4 | 3.8E-2 |
| GOTERM_BP_FAT | negative regulation of protein complex assembly | RT | 4 | 1.2E-3 | 5.4E-2 |
| GOTERM_BP_FAT | negative regulation of protein complex disassembly | RT | 4 | 2.1E-3 | 8.8E-2 |
| GOTERM_CC_FAT | intracellular non-membrane-bounded organelle | RT | 31 | 3.1E-3 | 4.6E-2 |
| GOTERM_CC_FAT | non-membrane-bounded organelle | RT | 31 | 3.1E-3 | 4.6E-2 |
| GOTERM_BP_FAT | regulation of protein complex disassembly | RT | 4 | 4.0E-3 | 1.5E-1 |
| GOTERM_BP_FAT | negative regulation of cytoskeleton organization | RT | 4 | 5.0E-3 | 1.7E-1 |
| GOTERM_BP_FAT | negative regulation of organelle organization | RT | 4 | 1.5E-2 | 3.7E-1 |
| KEGG_PATHWAY | Regulation of actin cytoskeleton | RT | 7 | 1.6E-2 | 2.9E-1 |
| KEGG_PATHWAY | Pathogenic Escherichia coli infection | RT | 4 | 1.7E-2 | 2.3E-1 |
| GOTERM_CC_FAT | cell cortex part | RT | 4 | 2.1E-2 | 1.6E-1 |
| GOTERM_CC_FAT | cytoskeleton | RT | 17 | 3.4E-2 | 2.1E-1 |
| GOTERM_CC_FAT | cytoskeletal part | RT | 13 | 3.7E-2 | 2.2E-1 |
| GOTERM_CC_FAT | cortical cytoskeleton | RT | 3 | 4.8E-2 | 2.5E-1 |
| GOTERM_BP_FAT | actin cytoskeleton organization | RT | 5 | 5.4E-2 | 6.4E-1 |
| GOTERM_BP_FAT | negative regulation of cellular component organization | RT | 4 | 6.0E-2 | 6.6E-1 |
| KEGG_PATHWAY | Fc gamma R-mediated phagocytosis | RT | 4 | 6.3E-2 | 5.5E-1 |
| GOTERM_BP_FAT | actin filament-based process | RT | 5 | 6.5E-2 | 6.8E-1 |
| GOTERM_CC_FAT | cell cortex | RT | 4 | 8.6E-2 | 3.7E-1 |
| GOTERM_BP_FAT | cell projection organization | RT | 5 | 2.0E-1 | 9.0E-1 |
| GOTERM_BP_FAT | cytoskeleton organization | RT | 5 | 2.9E-1 | 9.5E-1 |
| **Annotation Cluster 3** | **Enrichment Score: 2.51** | | **Count** | **P_Value** | **Benjamini** |
| GOTERM_CC_FAT | pigment granule | RT | 7 | 4.0E-5 | 7.9E-4 |
| GOTERM_CC_FAT | melanosome | RT | 7 | 4.0E-5 | 7.9E-4 |
| GOTERM_CC_FAT | cytoplasmic membrane-bounded vesicle | RT | 11 | 5.6E-3 | 7.2E-2 |
| GOTERM_CC_FAT | membrane-bounded vesicle | RT | 11 | 7.0E-3 | 8.3E-2 |
| GOTERM_CC_FAT | cytoplasmic vesicle | RT | 11 | 1.6E-2 | 1.6E-1 |
| GOTERM_CC_FAT | vesicle | RT | 11 | 2.1E-2 | 1.6E-1 |
| GOTERM_CC_FAT | secretory granule | RT | 4 | 1.4E-1 | 5.0E-1 |
| **Annotation Cluster 4** | **Enrichment Score: 2.08** | | **Count** | **P_Value** | **Benjamini** |
| GOTERM_CC_FAT | eukaryotic translation elongation factor 1 complex | RT | 3 | 4.9E-4 | 8.9E-3 |
| SP_PIR_KEYWORDS | elongation factor | RT | 3 | 5.3E-3 | 6.1E-2 |
| GOTERM_MF_FAT | translation elongation factor activity | RT | 3 | 1.2E-2 | 3.7E-1 |
| GOTERM_MF_FAT | translation factor activity, nucleic acid binding | RT | 3 | 1.5E-1 | 9.2E-1 |
| **Annotation Cluster 5** | **Enrichment Score: 2.02** | | **Count** | **P_Value** | **Benjamini** |
| GOTERM_MF_FAT | structural constituent of cytoskeleton | RT | 6 | 1.7E-4 | 1.1E-2 |
| SP_PIR_KEYWORDS | actin binding | RT | 4 | 1.2E-3 | 1.8E-2 |
| GOTERM_CC_FAT | sarcomere | RT | 4 | 3.3E-2 | 2.2E-1 |
| GOTERM_CC_FAT | myofibril | RT | 4 | 4.5E-2 | 2.5E-1 |
| GOTERM_CC_FAT | contractile fiber part | RT | 4 | 4.7E-2 | 2.5E-1 |
| GOTERM_CC_FAT | contractile fiber | RT | 4 | 5.5E-2 | 2.7E-1 |
| **Annotation Cluster 6** | **Enrichment Score: 1.85** | | **Count** | **P_Value** | **Benjamini** |
| SP_PIR_KEYWORDS | signal | RT | 31 | 8.2E-4 | 1.5E-2 |
| UP_SEQ_FEATURE | signal peptide | RT | 31 | 9.2E-4 | 1.9E-1 |
| SP_PIR_KEYWORDS | disulfide bond | RT | 27 | 3.5E-3 | 4.3E-2 |
| UP_SEQ_FEATURE | disulfide bond | RT | 26 | 4.8E-3 | 4.2E-1 |
| GOTERM_CC_FAT | extracellular region | RT | 23 | 2.3E-2 | 1.7E-1 |
| SP_PIR_KEYWORDS | Secreted | RT | 15 | 5.4E-2 | 3.3E-1 |
| SP_PIR_KEYWORDS | glycoprotein | RT | 27 | 2.3E-1 | 7.7E-1 |
| UP_SEQ_FEATURE | glycosylation site:N-linked (GlcNAc...) | RT | 24 | 4.1E-1 | 1.0E0 |
| **Annotation Cluster 7** | **Enrichment Score: 1.79** | | **Count** | **P_Value** | **Benjamini** |
| SMART | EGF_CA | RT | 5 | 1.2E-3 | 5.8E-2 |
| INTERPRO | [illegible] | RT | [illegible] | [illegible] | [illegible] |

| | | | | | |
|---|---|---|---|---|---|
| INTERPRO | EGF-like region, conserved site | RT | 8 | 1.6E-3 | 3.7E-1 |
| INTERPRO | EGF-like calcium-binding | RT | 5 | 2.4E-3 | 2.9E-1 |
| INTERPRO | EGF-like calcium-binding, conserved site | RT | 5 | 2.4E-3 | 2.9E-1 |
| UP_SEQ_FEATURE | domain:EGF-like 6; calcium-binding | RT | 3 | 7.5E-3 | 5.0E-1 |
| SP_PIR_KEYWORDS | extracellular matrix | RT | 6 | 8.5E-3 | 8.8E-2 |
| INTERPRO | EGF calcium-binding | RT | 4 | 8.5E-3 | 5.6E-1 |
| SP_PIR_KEYWORDS | calcium | RT | 11 | 8.9E-3 | 8.9E-2 |
| GOTERM_CC_FAT | extracellular matrix | RT | 8 | 1.1E-2 | 1.3E-1 |
| UP_SEQ_FEATURE | domain:EGF-like 4; calcium-binding | RT | 3 | 1.3E-2 | 6.3E-1 |
| UP_SEQ_FEATURE | domain:EGF-like 3; calcium-binding | RT | 3 | 1.6E-2 | 6.5E-1 |
| UP_SEQ_FEATURE | domain:EGF-like 5; calcium-binding | RT | 3 | 1.6E-2 | 6.5E-1 |
| GOTERM_CC_FAT | extracellular region part | RT | 14 | 1.8E-2 | 1.6E-1 |
| INTERPRO | EGF-type aspartate/asparagine hydroxylation conserved site | RT | 4 | 1.9E-2 | 7.5E-1 |
| GOTERM_MF_FAT | extracellular matrix structural constituent | RT | 4 | 2.2E-2 | 5.3E-1 |
| GOTERM_MF_FAT | calcium ion binding | RT | 13 | 2.6E-2 | 5.5E-1 |
| GOTERM_CC_FAT | proteinaceous extracellular matrix | RT | 7 | 2.7E-2 | 1.9E-1 |
| SP_PIR_KEYWORDS | egf-like domain | RT | 5 | 3.2E-2 | 2.3E-1 |
| SP_PIR_KEYWORDS | Secreted | RT | 15 | 5.4E-2 | 3.3E-1 |
| SMART | EGF | RT | 3 | 2.5E-1 | 9.8E-1 |
| INTERPRO | EGF-like, type 3 | RT | 3 | 3.1E-1 | 1.0E0 |
| INTERPRO | EGF-like | RT | 3 | 3.3E-1 | 1.0E0 |
| **Annotation Cluster 8** | **Enrichment Score: 1.77** | | **Count** | **P_Value** | **Benjamini** |
| UP_SEQ_FEATURE | short sequence motif:Prevents secretion from ER | RT | 5 | 2.6E-4 | 1.1E-1 |
| GOTERM_CC_FAT | endoplasmic reticulum lumen | RT | 4 | 1.9E-2 | 1.6E-1 |
| SP_PIR_KEYWORDS | endoplasmic reticulum | RT | 9 | 3.2E-2 | 2.3E-1 |
| GOTERM_CC_FAT | endoplasmic reticulum | RT | 13 | 3.9E-2 | 2.3E-1 |
| GOTERM_CC_FAT | endoplasmic reticulum part | RT | 5 | 2.3E-1 | 6.7E-1 |
| **Annotation Cluster 9** | **Enrichment Score: 1.64** | | **Count** | **P_Value** | **Benjamini** |
| SP_PIR_KEYWORDS | nadp | RT | 6 | 1.3E-3 | 2.0E-2 |
| GOTERM_MF_FAT | oxidoreductase activity, acting on the CH-CH group of donors, NAD or NADP as acceptor | RT | 3 | 7.7E-3 | 2.9E-1 |
| SP_PIR_KEYWORDS | oxidoreductase | RT | 9 | 9.0E-3 | 8.5E-2 |
| GOTERM_BP_FAT | oxidation reduction | RT | 10 | 1.9E-2 | 4.0E-1 |
| INTERPRO | NAD(P)-binding domain | RT | 4 | 5.7E-2 | 9.4E-1 |
| GOTERM_MF_FAT | cofactor binding | RT | 5 | 9.7E-2 | 8.4E-1 |
| GOTERM_MF_FAT | coenzyme binding | RT | 3 | 3.6E-1 | 9.9E-1 |
| **Annotation Cluster 10** | **Enrichment Score: 1.42** | | **Count** | **P_Value** | **Benjamini** |
| SP_PIR_KEYWORDS | plasma | RT | 5 | 1.4E-3 | 2.0E-2 |
| SP_PIR_KEYWORDS | duplication | RT | 6 | 6.8E-3 | 7.4E-2 |
| GOTERM_BP_FAT | acute inflammatory response | RT | 4 | 2.4E-2 | 4.6E-1 |
| GOTERM_BP_FAT | response to wounding | RT | 7 | 1.2E-1 | 8.0E-1 |
| GOTERM_BP_FAT | inflammatory response | RT | 4 | 3.3E-1 | 9.6E-1 |
| GOTERM_BP_FAT | defense response | RT | 6 | 3.4E-1 | 9.6E-1 |
| **Annotation Cluster 11** | **Enrichment Score: 1.36** | | **Count** | **P_Value** | **Benjamini** |
| SP_PIR_KEYWORDS | gangliosidosis | RT | 3 | 2.6E-4 | 5.7E-3 |
| GOTERM_BP_FAT | glycosphingolipid metabolic process | RT | 4 | 5.6E-4 | 3.4E-2 |
| GOTERM_BP_FAT | glycolipid metabolic process | RT | 4 | 9.5E-4 | 4.4E-2 |
| SP_PIR_KEYWORDS | lysosome | RT | 6 | 1.1E-3 | 1.8E-2 |
| KEGG_PATHWAY | Lysosome | RT | 6 | 5.0E-3 | 1.4E-1 |
| GOTERM_BP_FAT | sphingolipid metabolic process | RT | 4 | 1.2E-2 | 3.2E-1 |
| GOTERM_BP_FAT | membrane lipid metabolic process | RT | 4 | 1.4E-2 | 3.7E-1 |
| GOTERM_CC_FAT | lysosome | RT | 6 | 1.7E-2 | 1.6E-1 |
| GOTERM_CC_FAT | lytic vacuole | RT | 6 | 1.7E-2 | 1.6E-1 |
| SP_PIR_KEYWORDS | zymogen | RT | 5 | 2.3E-2 | 1.8E-1 |
| GOTERM_CC_FAT | vacuole | RT | 6 | 3.4E-2 | 2.1E-1 |
| UP_SEQ_FEATURE | propeptide:Activation peptide | RT | 3 | 5.6E-2 | 9.5E-1 |
| GOTERM_MF_FAT | enzyme activator activity | RT | 5 | 2.1E-1 | 9.6E-1 |
| SP_PIR_KEYWORDS | Protease | RT | 5 | 2.4E-1 | 7.9E-1 |

| | | | | | |
|---|---|---|---|---|---|
| GOTERM_BP_FAT | lipid localization | RT | 3 | 2.6E-1 | 9.3E-1 |
| SP_PIR_KEYWORDS | hydrolase | RT | 11 | 2.9E-1 | 8.4E-1 |
| GOTERM_BP_FAT | proteolysis | RT | 9 | 3.4E-1 | 9.6E-1 |
| GOTERM_MF_FAT | serine-type peptidase activity | RT | 3 | 3.6E-1 | 9.9E-1 |
| GOTERM_MF_FAT | serine hydrolase activity | RT | 3 | 3.6E-1 | 9.9E-1 |
| GOTERM_MF_FAT | endopeptidase activity | RT | 4 | 4.9E-1 | 1.0E0 |
| GOTERM_MF_FAT | peptidase activity, acting on L-amino acid peptides | RT | 5 | 5.4E-1 | 1.0E0 |
| GOTERM_MF_FAT | peptidase activity | RT | 5 | 5.8E-1 | 1.0E0 |
| GOTERM_CC_FAT | integral to membrane | RT | 7 | 1.0E0 | 1.0E0 |
| **Annotation Cluster 12** | **Enrichment Score: 1.34** | | **Count** | **P_Value** | **Benjamini** |
| GOTERM_BP_FAT | NADP metabolic process | RT | 3 | 4.4E-3 | 1.6E-1 |
| GOTERM_BP_FAT | nicotinamide nucleotide metabolic process | RT | 3 | 2.6E-2 | 4.9E-1 |
| GOTERM_BP_FAT | nicotinamide metabolic process | RT | 3 | 2.6E-2 | 4.9E-1 |
| GOTERM_BP_FAT | alkaloid metabolic process | RT | 3 | 2.7E-2 | 4.9E-1 |
| GOTERM_BP_FAT | pyridine nucleotide metabolic process | RT | 3 | 2.9E-2 | 4.9E-1 |
| GOTERM_BP_FAT | oxidoreduction coenzyme metabolic process | RT | 3 | 4.2E-2 | 6.0E-1 |
| GOTERM_BP_FAT | cellular amide metabolic process | RT | 3 | 4.8E-2 | 6.2E-1 |
| GOTERM_BP_FAT | secondary metabolic process | RT | 3 | 8.8E-2 | 7.3E-1 |
| GOTERM_BP_FAT | coenzyme metabolic process | RT | 3 | 2.5E-1 | 9.3E-1 |
| GOTERM_BP_FAT | cofactor metabolic process | RT | 3 | 3.5E-1 | 9.6E-1 |
| **Annotation Cluster 13** | **Enrichment Score: 1.19** | | **Count** | **P_Value** | **Benjamini** |
| GOTERM_BP_FAT | cellular response to reactive oxygen species | RT | 3 | 1.5E-2 | 3.7E-1 |
| GOTERM_BP_FAT | cellular response to oxidative stress | RT | 3 | 3.0E-2 | 4.9E-1 |
| GOTERM_BP_FAT | cellular response to stress | RT | 8 | 6.5E-2 | 6.8E-1 |
| GOTERM_BP_FAT | response to reactive oxygen species | RT | 3 | 8.1E-2 | 7.1E-1 |
| GOTERM_BP_FAT | response to oxidative stress | RT | 4 | 8.4E-2 | 7.2E-1 |
| GOTERM_BP_FAT | response to inorganic substance | RT | 3 | 3.7E-1 | 9.7E-1 |
| **Annotation Cluster 14** | **Enrichment Score: 1.19** | | **Count** | **P_Value** | **Benjamini** |
| GOTERM_BP_FAT | cell motion | RT | 11 | 7.7E-4 | 3.9E-2 |
| GOTERM_BP_FAT | cell migration | RT | 4 | 2.5E-1 | 9.3E-1 |
| GOTERM_BP_FAT | localization of cell | RT | 4 | 3.0E-1 | 9.5E-1 |
| GOTERM_BP_FAT | cell motility | RT | 4 | 3.0E-1 | 9.5E-1 |
| **Annotation Cluster 15** | **Enrichment Score: 1.19** | | **Count** | **P_Value** | **Benjamini** |
| GOTERM_BP_FAT | gonad development | RT | 4 | 3.3E-2 | 5.2E-1 |
| GOTERM_BP_FAT | reproductive structure development | RT | 4 | 4.5E-2 | 6.1E-1 |
| GOTERM_BP_FAT | development of primary sexual characteristics | RT | 4 | 4.6E-2 | 6.0E-1 |
| GOTERM_BP_FAT | female gonad development | RT | 3 | 6.1E-2 | 6.7E-1 |
| GOTERM_BP_FAT | sex differentiation | RT | 4 | 7.0E-2 | 6.9E-1 |
| GOTERM_BP_FAT | female sex differentiation | RT | 3 | 7.0E-2 | 6.9E-1 |
| GOTERM_BP_FAT | development of primary female sexual characteristics | RT | 3 | 7.0E-2 | 6.9E-1 |
| GOTERM_BP_FAT | reproductive developmental process | RT | 4 | 2.3E-1 | 9.1E-1 |
| **Annotation Cluster 16** | **Enrichment Score: 1.12** | | **Count** | **P_Value** | **Benjamini** |
| GOTERM_MF_FAT | enzyme inhibitor activity | RT | 6 | 4.1E-2 | 6.7E-1 |
| GOTERM_MF_FAT | endopeptidase inhibitor activity | RT | 4 | 8.2E-2 | 8.5E-1 |
| GOTERM_MF_FAT | peptidase inhibitor activity | RT | 4 | 9.2E-2 | 8.6E-1 |
| SP_PIR_KEYWORDS | protease inhibitor | RT | 3 | 1.1E-1 | 5.2E-1 |
| **Annotation Cluster 17** | **Enrichment Score: 1.03** | | **Count** | **P_Value** | **Benjamini** |
| SP_PIR_KEYWORDS | isopeptide bond | RT | 6 | 2.6E-2 | 2.0E-1 |
| UP_SEQ_FEATURE | cross-link:Glycyl lysine isopeptide (Lys-Gly) (interchain with G-Cter in ubiquitin) | RT | 4 | 8.7E-2 | 9.7E-1 |
| SP_PIR_KEYWORDS | ubl conjugation | RT | 5 | 3.7E-1 | 9.1E-1 |
| **Annotation Cluster 18** | **Enrichment Score: 1.03** | | **Count** | **P_Value** | **Benjamini** |
| GOTERM_BP_FAT | regulation of DNA repair | RT | 3 | 8.3E-3 | 2.5E-1 |
| GOTERM_BP_FAT | regulation of cellular response to stress | RT | 4 | 2.7E-2 | 4.9E-1 |
| GOTERM_BP_FAT | regulation of binding | RT | 4 | 7.2E-2 | 6.9E-1 |
| GOTERM_BP_FAT | regulation of transcription factor activity | RT | 3 | 1.4E-1 | 8.3E-1 |
| GOTERM_BP_FAT | regulation of DNA metabolic process | RT | 3 | 1.6E-1 | 8.7E-1 |
| GOTERM_BP_FAT | regulation of DNA binding | RT | 3 | 1.8E-1 | 8.8E-1 |

| | | | | | |
|---|---|---|---|---|---|
| GOTERM_BP_FAT | regulation of transcription | RT | 3 | 1.0E0 | 1.0E0 |
| **Annotation Cluster 19** | **Enrichment Score: 0.97** | | **Count** | **P_Value** | **Benjamini** |
| INTERPRO | EF-Hand type | RT | 5 | 4.9E-2 | 9.5E-1 |
| UP_SEQ_FEATURE | domain:EF-hand 1 | RT | 4 | 7.0E-2 | 9.5E-1 |
| UP_SEQ_FEATURE | domain:EF-hand 2 | RT | 4 | 7.0E-2 | 9.5E-1 |
| UP_SEQ_FEATURE | domain:EF-hand 3 | RT | 3 | 8.7E-2 | 9.6E-1 |
| UP_SEQ_FEATURE | calcium-binding region:2 | RT | 3 | 1.2E-1 | 9.9E-1 |
| INTERPRO | EF-HAND 2 | RT | 4 | 1.3E-1 | 9.9E-1 |
| INTERPRO | EF-HAND 1 | RT | 4 | 1.3E-1 | 9.8E-1 |
| UP_SEQ_FEATURE | calcium-binding region:1 | RT | 3 | 1.4E-1 | 9.9E-1 |
| SMART | EFh | RT | 3 | 1.5E-1 | 9.4E-1 |
| INTERPRO | Calcium-binding EF-hand | RT | 3 | 2.0E-1 | 1.0E0 |
| **Annotation Cluster 20** | **Enrichment Score: 0.95** | | **Count** | **P_Value** | **Benjamini** |
| INTERPRO | EGF-like region, conserved site | RT | 8 | 1.6E-3 | 3.7E-1 |
| SP_PIR_KEYWORDS | extracellular matrix | RT | 6 | 8.5E-3 | 8.8E-2 |
| GOTERM_CC_FAT | extracellular matrix | RT | 8 | 1.1E-2 | 1.3E-1 |
| GOTERM_CC_FAT | proteinaceous extracellular matrix | RT | 7 | 2.7E-2 | 1.9E-1 |
| GOTERM_CC_FAT | basement membrane | RT | 3 | 1.1E-1 | 4.2E-1 |
| KEGG_PATHWAY | Small cell lung cancer | RT | 3 | 1.9E-1 | 8.9E-1 |
| KEGG_PATHWAY | ECM-receptor interaction | RT | 3 | 1.9E-1 | 8.9E-1 |
| GOTERM_CC_FAT | extracellular matrix part | RT | 3 | 2.0E-1 | 6.4E-1 |
| KEGG_PATHWAY | Pathways in cancer | RT | 5 | 3.9E-1 | 9.9E-1 |
| KEGG_PATHWAY | Focal adhesion | RT | 3 | 5.8E-1 | 1.0E0 |
| SP_PIR_KEYWORDS | cell adhesion | RT | 3 | 6.5E-1 | 9.9E-1 |
| GOTERM_BP_FAT | cell adhesion | RT | 3 | 9.4E-1 | 1.0E0 |
| GOTERM_BP_FAT | biological adhesion | RT | 3 | 9.4E-1 | 1.0E0 |
| **Annotation Cluster 21** | **Enrichment Score: 0.92** | | **Count** | **P_Value** | **Benjamini** |
| GOTERM_CC_FAT | organelle lumen | RT | 19 | 9.0E-2 | 3.8E-1 |
| GOTERM_CC_FAT | membrane-enclosed lumen | RT | 19 | 1.0E-1 | 4.2E-1 |
| GOTERM_CC_FAT | intracellular organelle lumen | RT | 17 | 1.9E-1 | 6.2E-1 |
| **Annotation Cluster 22** | **Enrichment Score: 0.89** | | **Count** | **P_Value** | **Benjamini** |
| GOTERM_BP_FAT | positive regulation of hydrolase activity | RT | 6 | 5.4E-3 | 1.8E-1 |
| GOTERM_BP_FAT | regulation of peptidase activity | RT | 4 | 1.7E-2 | 3.8E-1 |
| GOTERM_BP_FAT | regulation of hydrolase activity | RT | 7 | 1.9E-2 | 4.1E-1 |
| GOTERM_BP_FAT | activation of caspase activity | RT | 3 | 4.5E-2 | 6.1E-1 |
| GOTERM_BP_FAT | positive regulation of peptidase activity | RT | 3 | 5.3E-2 | 6.4E-1 |
| GOTERM_BP_FAT | positive regulation of caspase activity | RT | 3 | 5.3E-2 | 6.4E-1 |
| GOTERM_BP_FAT | regulation of caspase activity | RT | 3 | 8.8E-2 | 7.3E-1 |
| GOTERM_BP_FAT | regulation of endopeptidase activity | RT | 3 | 9.4E-2 | 7.4E-1 |
| GOTERM_BP_FAT | regulation of apoptosis | RT | 7 | 3.9E-1 | 9.7E-1 |
| GOTERM_BP_FAT | regulation of programmed cell death | RT | 7 | 4.0E-1 | 9.7E-1 |
| GOTERM_BP_FAT | regulation of cell death | RT | 7 | 4.1E-1 | 9.7E-1 |
| GOTERM_BP_FAT | cell death | RT | 5 | 6.7E-1 | 1.0E0 |
| GOTERM_BP_FAT | death | RT | 5 | 6.7E-1 | 1.0E0 |
| GOTERM_BP_FAT | apoptosis | RT | 3 | 9.0E-1 | 1.0E0 |
| GOTERM_BP_FAT | programmed cell death | RT | 3 | 9.0E-1 | 1.0E0 |
| **Annotation Cluster 23** | **Enrichment Score: 0.73** | | **Count** | **P_Value** | **Benjamini** |
| SP_PIR_KEYWORDS | calcium | RT | 11 | 8.9E-3 | 8.9E-2 |
| GOTERM_MF_FAT | calcium ion binding | RT | 13 | 2.6E-2 | 5.5E-1 |
| GOTERM_MF_FAT | cation binding | RT | 21 | 9.9E-1 | 1.0E0 |
| GOTERM_MF_FAT | ion binding | RT | 21 | 9.9E-1 | 1.0E0 |
| GOTERM_MF_FAT | metal ion binding | RT | 20 | 9.9E-1 | 1.0E0 |
| **Annotation Cluster 24** | **Enrichment Score: 0.64** | | **Count** | **P_Value** | **Benjamini** |
| GOTERM_BP_FAT | cellular homeostasis | RT | 7 | 7.3E-2 | 6.9E-1 |
| GOTERM_BP_FAT | cellular cation homeostasis | RT | 5 | 7.6E-2 | 7.0E-1 |
| GOTERM_BP_FAT | cation homeostasis | RT | 5 | 1.1E-1 | 7.7E-1 |
| GOTERM_BP_FAT | cellular di-, tri-valent inorganic cation homeostasis | RT | 4 | 1.7E-1 | 8.8E-1 |
| GOTERM_BP_FAT | di-, tri-valent inorganic cation homeostasis | RT | 4 | 1.9E-1 | 8.9E-1 |

| Category | Term | RT | Count | P_Value | Benjamini |
|---|---|---|---|---|---|
| GOTERM_BP_FAT | cellular ion homeostasis | RT | 5 | 2.1E-1 | 9.0E-1 |
| GOTERM_BP_FAT | cellular chemical homeostasis | RT | 5 | 2.2E-1 | 9.0E-1 |
| GOTERM_BP_FAT | membrane organization | RT | 5 | 2.2E-1 | 9.0E-1 |
| GOTERM_BP_FAT | ion homeostasis | RT | 5 | 2.6E-1 | 9.3E-1 |
| GOTERM_BP_FAT | cellular calcium ion homeostasis | RT | 3 | 3.2E-1 | 9.5E-1 |
| GOTERM_BP_FAT | calcium ion homeostasis | RT | 3 | 3.3E-1 | 9.6E-1 |
| GOTERM_BP_FAT | homeostatic process | RT | 7 | 3.3E-1 | 9.6E-1 |
| GOTERM_BP_FAT | cellular metal ion homeostasis | RT | 3 | 3.5E-1 | 9.6E-1 |
| GOTERM_BP_FAT | metal ion homeostasis | RT | 3 | 3.7E-1 | 9.7E-1 |
| GOTERM_BP_FAT | chemical homeostasis | RT | 5 | 4.0E-1 | 9.7E-1 |
| GOTERM_BP_FAT | cell proliferation | RT | 3 | 7.6E-1 | 1.0E0 |
| **Annotation Cluster 25** | **Enrichment Score: 0.61** | | **Count** | **P_Value** | **Benjamini** |
| GOTERM_MF_FAT | vitamin binding | RT | 3 | 2.3E-1 | 9.6E-1 |
| GOTERM_BP_FAT | carboxylic acid biosynthetic process | RT | 3 | 2.5E-1 | 9.3E-1 |
| GOTERM_BP_FAT | organic acid biosynthetic process | RT | 3 | 2.5E-1 | 9.3E-1 |
| **Annotation Cluster 26** | **Enrichment Score: 0.6** | | **Count** | **P_Value** | **Benjamini** |
| GOTERM_BP_FAT | positive regulation of molecular function | RT | 10 | 1.1E-2 | 3.2E-1 |
| GOTERM_BP_FAT | positive regulation of catalytic activity | RT | 9 | 1.6E-2 | 3.8E-1 |
| GOTERM_BP_FAT | positive regulation of ubiquitin-protein ligase activity | RT | 3 | 7.2E-2 | 6.9E-1 |
| GOTERM_BP_FAT | positive regulation of ligase activity | RT | 3 | 7.7E-2 | 7.0E-1 |
| GOTERM_BP_FAT | regulation of ubiquitin-protein ligase activity | RT | 3 | 8.6E-2 | 7.2E-1 |
| GOTERM_BP_FAT | regulation of ligase activity | RT | 3 | 9.2E-2 | 7.4E-1 |
| GOTERM_BP_FAT | positive regulation of protein ubiquitination | RT | 3 | 9.8E-2 | 7.5E-1 |
| GOTERM_BP_FAT | regulation of protein ubiquitination | RT | 3 | 1.3E-1 | 8.2E-1 |
| GOTERM_BP_FAT | negative regulation of protein modification process | RT | 3 | 1.7E-1 | 8.7E-1 |
| GOTERM_BP_FAT | regulation of protein modification process | RT | 4 | 2.8E-1 | 9.4E-1 |
| GOTERM_BP_FAT | negative regulation of cellular protein metabolic process | RT | 3 | 3.1E-1 | 9.5E-1 |
| GOTERM_BP_FAT | negative regulation of protein metabolic process | RT | 3 | 3.3E-1 | 9.6E-1 |
| GOTERM_BP_FAT | positive regulation of protein modification process | RT | 3 | 3.3E-1 | 9.6E-1 |
| GOTERM_BP_FAT | proteolysis | RT | 9 | 3.4E-1 | 9.6E-1 |
| GOTERM_BP_FAT | positive regulation of cellular protein metabolic process | RT | 3 | 4.3E-1 | 9.8E-1 |
| GOTERM_BP_FAT | positive regulation of protein metabolic process | RT | 3 | 4.5E-1 | 9.8E-1 |
| GOTERM_BP_FAT | cellular macromolecule catabolic process | RT | 6 | 4.8E-1 | 9.9E-1 |
| GOTERM_BP_FAT | macromolecule catabolic process | RT | 6 | 5.5E-1 | 9.9E-1 |
| GOTERM_BP_FAT | regulation of cellular protein metabolic process | RT | 4 | 5.8E-1 | 1.0E0 |
| GOTERM_BP_FAT | modification-dependent protein catabolic process | RT | 4 | 7.1E-1 | 1.0E0 |
| GOTERM_BP_FAT | modification-dependent macromolecule catabolic process | RT | 4 | 7.1E-1 | 1.0E0 |
| GOTERM_BP_FAT | proteolysis involved in cellular protein catabolic process | RT | 4 | 7.3E-1 | 1.0E0 |
| GOTERM_BP_FAT | cellular protein catabolic process | RT | 4 | 7.4E-1 | 1.0E0 |
| GOTERM_BP_FAT | protein catabolic process | RT | 4 | 7.6E-1 | 1.0E0 |
| GOTERM_BP_FAT | negative regulation of macromolecule metabolic process | RT | 4 | 8.5E-1 | 1.0E0 |
| GOTERM_BP_FAT | positive regulation of macromolecule metabolic process | RT | 3 | 9.7E-1 | 1.0E0 |
| **Annotation Cluster 27** | **Enrichment Score: 0.59** | | **Count** | **P_Value** | **Benjamini** |
| GOTERM_BP_FAT | intracellular transport | RT | 9 | 5.4E-2 | 6.4E-1 |
| GOTERM_BP_FAT | protein localization | RT | 9 | 1.9E-1 | 8.9E-1 |
| GOTERM_BP_FAT | protein transport | RT | 8 | 2.0E-1 | 9.0E-1 |
| GOTERM_BP_FAT | establishment of protein localization | RT | 8 | 2.1E-1 | 9.0E-1 |
| GOTERM_BP_FAT | cellular protein localization | RT | 5 | 2.6E-1 | 9.3E-1 |
| GOTERM_BP_FAT | cellular macromolecule localization | RT | 5 | 2.6E-1 | 9.3E-1 |
| GOTERM_BP_FAT | intracellular protein transport | RT | 4 | 4.2E-1 | 9.8E-1 |
| SP_PIR_KEYWORDS | protein transport | RT | 4 | 4.6E-1 | 9.5E-1 |
| SP_PIR_KEYWORDS | transport | RT | 6 | 9.4E-1 | 1.0E0 |
| **Annotation Cluster 28** | **Enrichment Score: 0.59** | | **Count** | **P_Value** | **Benjamini** |

| | | | | | | | |
|---|---|---|---|---|---|---|---|
| | GOTERM_MF_FAT | identical protein binding | RT | | 9 | 8.0E-2 | 8.7E-1 |
| | GOTERM_MF_FAT | protein homodimerization activity | RT | | 4 | 4.2E-1 | 9.9E-1 |
| | GOTERM_MF_FAT | protein dimerization activity | RT | | 5 | 5.3E-1 | 1.0E0 |
| | Annotation Cluster 29 | Enrichment Score: 0.57 | | | Count | P_Value | Benjamini |
| | GOTERM_BP_FAT | positive regulation of I-kappaB kinase/NF-kappaB cascade | RT | | 3 | 1.2E-1 | 8.1E-1 |
| | GOTERM_BP_FAT | regulation of I-kappaB kinase/NF-kappaB cascade | RT | | 3 | 1.5E-1 | 8.5E-1 |
| | GOTERM_BP_FAT | regulation of protein kinase cascade | RT | | 4 | 2.1E-1 | 9.0E-1 |
| | GOTERM_BP_FAT | positive regulation of protein kinase cascade | RT | | 3 | 2.8E-1 | 9.4E-1 |
| | GOTERM_BP_FAT | positive regulation of signal transduction | RT | | 3 | 5.6E-1 | 9.9E-1 |
| | GOTERM_BP_FAT | positive regulation of cell communication | RT | | 3 | 6.2E-1 | 1.0E0 |
| | Annotation Cluster 30 | Enrichment Score: 0.57 | | | Count | P_Value | Benjamini |
| | GOTERM_BP_FAT | activation of immune response | RT | | 3 | 1.2E-1 | 8.0E-1 |
| | GOTERM_BP_FAT | positive regulation of immune response | RT | | 3 | 2.3E-1 | 9.1E-1 |
| | GOTERM_BP_FAT | positive regulation of response to stimulus | RT | | 3 | 4.4E-1 | 9.8E-1 |
| | GOTERM_BP_FAT | positive regulation of immune system process | RT | | 3 | 4.4E-1 | 9.8E-1 |
| | Annotation Cluster 31 | Enrichment Score: 0.48 | | | Count | P_Value | Benjamini |
| | GOTERM_BP_FAT | immune effector process | RT | | 3 | 2.1E-1 | 9.0E-1 |
| | GOTERM_BP_FAT | innate immune response | RT | | 3 | 2.2E-1 | 9.0E-1 |
| | GOTERM_BP_FAT | defense response | RT | | 6 | 3.4E-1 | 9.6E-1 |
| | GOTERM_BP_FAT | immune response | RT | | 4 | 8.2E-1 | 1.0E0 |
| | Annotation Cluster 32 | Enrichment Score: 0.44 | | | Count | P_Value | Benjamini |
| | SP_PIR_KEYWORDS | ATP | RT | | 4 | 1.2E-1 | 5.6E-1 |
| | GOTERM_BP_FAT | ATP metabolic process | RT | | 3 | 1.4E-1 | 8.4E-1 |
| | GOTERM_BP_FAT | purine ribonucleoside triphosphate metabolic process | RT | | 3 | 1.7E-1 | 8.7E-1 |
| | GOTERM_BP_FAT | ribonucleoside triphosphate metabolic process | RT | | 3 | 1.7E-1 | 8.8E-1 |
| | GOTERM_BP_FAT | purine nucleoside triphosphate metabolic process | RT | | 3 | 1.8E-1 | 8.8E-1 |
| | GOTERM_BP_FAT | nucleoside triphosphate metabolic process | RT | | 3 | 2.0E-1 | 9.0E-1 |
| | GOTERM_BP_FAT | purine ribonucleotide metabolic process | RT | | 3 | 2.2E-1 | 9.0E-1 |
| | GOTERM_CC_FAT | mitochondrial matrix | RT | | 4 | 2.2E-1 | 6.6E-1 |
| | GOTERM_CC_FAT | mitochondrial lumen | RT | | 4 | 2.2E-1 | 6.6E-1 |
| | GOTERM_BP_FAT | ribonucleotide metabolic process | RT | | 3 | 2.4E-1 | 9.2E-1 |
| | GOTERM_BP_FAT | purine nucleotide metabolic process | RT | | 3 | 3.3E-1 | 9.6E-1 |
| | SP_PIR_KEYWORDS | nucleotide-binding | RT | | 11 | 3.8E-1 | 9.2E-1 |
| | GOTERM_CC_FAT | mitochondrial part | RT | | 6 | 4.2E-1 | 8.8E-1 |
| | SP_PIR_KEYWORDS | ligase | RT | | 3 | 4.7E-1 | 9.5E-1 |
| | SP_PIR_KEYWORDS | atp-binding | RT | | 6 | 8.3E-1 | 1.0E0 |
| | UP_SEQ_FEATURE | nucleotide phosphate-binding region:ATP | RT | | 4 | 8.9E-1 | 1.0E0 |
| | GOTERM_MF_FAT | ATP binding | RT | | 8 | 9.1E-1 | 1.0E0 |
| | GOTERM_MF_FAT | adenyl ribonucleotide binding | RT | | 8 | 9.1E-1 | 1.0E0 |
| | GOTERM_MF_FAT | adenyl nucleotide binding | RT | | 8 | 9.4E-1 | 1.0E0 |
| | GOTERM_MF_FAT | purine nucleoside binding | RT | | 8 | 9.4E-1 | 1.0E0 |
| | GOTERM_MF_FAT | nucleoside binding | RT | | 8 | 9.5E-1 | 1.0E0 |
| | Annotation Cluster 33 | Enrichment Score: 0.38 | | | Count | P_Value | Benjamini |
| | GOTERM_BP_FAT | cellular component morphogenesis | RT | | 5 | 2.4E-1 | 9.2E-1 |
| | GOTERM_BP_FAT | cell morphogenesis involved in differentiation | RT | | 3 | 4.6E-1 | 9.8E-1 |
| | GOTERM_BP_FAT | cell morphogenesis | RT | | 3 | 6.6E-1 | 1.0E0 |
| | Annotation Cluster 34 | Enrichment Score: 0.36 | | | Count | P_Value | Benjamini |
| | GOTERM_BP_FAT | response to organic substance | RT | | 8 | 1.7E-1 | 8.8E-1 |
| | GOTERM_BP_FAT | response to hormone stimulus | RT | | 3 | 6.8E-1 | 1.0E0 |
| | GOTERM_BP_FAT | response to endogenous stimulus | RT | | 3 | 7.3E-1 | 1.0E0 |
| | Annotation Cluster 35 | Enrichment Score: 0.35 | | | Count | P_Value | Benjamini |
| | GOTERM_BP_FAT | cellular response to stress | RT | | 8 | 6.5E-2 | 6.8E-1 |
| | GOTERM_BP_FAT | regulation of apoptosis | RT | | 7 | 3.9E-1 | 9.7E-1 |
| | GOTERM_BP_FAT | regulation of programmed cell death | RT | | 7 | 4.0E-1 | 9.7E-1 |
| | GOTERM_BP_FAT | regulation of cell death | RT | | 7 | 4.1E-1 | 9.7E-1 |
| | GOTERM_BP_FAT | induction of apoptosis | RT | | 3 | 6.0E-1 | 1.0E0 |

| Category | Term | RT | Count | P_Value | Benjamini |
|---|---|---|---|---|---|
| GOTERM_BP_FAT | induction of programmed cell death | RT | 3 | 6.0E-1 | 1.0E0 |
| GOTERM_BP_FAT | positive regulation of apoptosis | RT | 3 | 7.6E-1 | 1.0E0 |
| GOTERM_BP_FAT | positive regulation of programmed cell death | RT | 3 | 7.6E-1 | 1.0E0 |
| GOTERM_BP_FAT | positive regulation of cell death | RT | 3 | 7.6E-1 | 1.0E0 |
| **Annotation Cluster 36** | **Enrichment Score: 0.32** | | **Count** | **P_Value** | **Benjamini** |
| UP_SEQ_FEATURE | nucleotide phosphate-binding region:GTP | RT | 5 | 6.9E-2 | 9.6E-1 |
| SP_PIR_KEYWORDS | gtp-binding | RT | 5 | 9.3E-2 | 4.8E-1 |
| GOTERM_MF_FAT | GTPase activity | RT | 4 | 1.8E-1 | 9.4E-1 |
| GOTERM_MF_FAT | GTP binding | RT | 5 | 2.6E-1 | 9.7E-1 |
| GOTERM_MF_FAT | guanyl nucleotide binding | RT | 5 | 2.8E-1 | 9.7E-1 |
| GOTERM_MF_FAT | guanyl ribonucleotide binding | RT | 5 | 2.8E-1 | 9.7E-1 |
| SP_PIR_KEYWORDS | nucleotide-binding | RT | 11 | 3.8E-1 | 9.2E-1 |
| GOTERM_MF_FAT | ribonucleotide binding | RT | 12 | 7.6E-1 | 1.0E0 |
| GOTERM_MF_FAT | purine ribonucleotide binding | RT | 12 | 7.6E-1 | 1.0E0 |
| GOTERM_MF_FAT | purine nucleotide binding | RT | 12 | 8.1E-1 | 1.0E0 |
| GOTERM_MF_FAT | nucleotide binding | RT | 14 | 8.1E-1 | 1.0E0 |
| SP_PIR_KEYWORDS | atp-binding | RT | 6 | 8.3E-1 | 1.0E0 |
| UP_SEQ_FEATURE | nucleotide phosphate-binding region:ATP | RT | 4 | 8.9E-1 | 1.0E0 |
| GOTERM_MF_FAT | ATP binding | RT | 8 | 9.1E-1 | 1.0E0 |
| GOTERM_MF_FAT | adenyl ribonucleotide binding | RT | 8 | 9.1E-1 | 1.0E0 |
| GOTERM_MF_FAT | adenyl nucleotide binding | RT | 8 | 9.4E-1 | 1.0E0 |
| GOTERM_MF_FAT | purine nucleoside binding | RT | 8 | 9.4E-1 | 1.0E0 |
| GOTERM_MF_FAT | nucleoside binding | RT | 8 | 9.5E-1 | 1.0E0 |
| **Annotation Cluster 37** | **Enrichment Score: 0.31** | | **Count** | **P_Value** | **Benjamini** |
| GOTERM_BP_FAT | anti-apoptosis | RT | 3 | 3.7E-1 | 9.7E-1 |
| GOTERM_BP_FAT | regulation of apoptosis | RT | 7 | 3.9E-1 | 9.7E-1 |
| GOTERM_BP_FAT | regulation of programmed cell death | RT | 7 | 4.0E-1 | 9.7E-1 |
| GOTERM_BP_FAT | regulation of cell death | RT | 7 | 4.1E-1 | 9.7E-1 |
| GOTERM_BP_FAT | negative regulation of apoptosis | RT | 3 | 6.6E-1 | 1.0E0 |
| GOTERM_BP_FAT | negative regulation of programmed cell death | RT | 3 | 6.6E-1 | 1.0E0 |
| GOTERM_BP_FAT | negative regulation of cell death | RT | 3 | 6.7E-1 | 1.0E0 |
| **Annotation Cluster 38** | **Enrichment Score: 0.3** | | **Count** | **P_Value** | **Benjamini** |
| GOTERM_CC_FAT | mitochondrion | RT | 13 | 8.4E-2 | 3.7E-1 |
| GOTERM_CC_FAT | mitochondrial part | RT | 6 | 4.2E-1 | 8.8E-1 |
| GOTERM_CC_FAT | nuclear envelope | RT | 3 | 4.3E-1 | 8.9E-1 |
| SP_PIR_KEYWORDS | mitochondrion | RT | 6 | 4.4E-1 | 9.4E-1 |
| GOTERM_CC_FAT | organelle envelope | RT | 6 | 4.5E-1 | 9.0E-1 |
| GOTERM_CC_FAT | envelope | RT | 6 | 4.6E-1 | 9.0E-1 |
| GOTERM_CC_FAT | organelle inner membrane | RT | 3 | 6.8E-1 | 9.8E-1 |
| GOTERM_CC_FAT | mitochondrial membrane | RT | 3 | 7.8E-1 | 9.9E-1 |
| GOTERM_CC_FAT | mitochondrial envelope | RT | 3 | 8.0E-1 | 9.9E-1 |
| GOTERM_CC_FAT | endomembrane system | RT | 5 | 8.2E-1 | 1.0E0 |
| GOTERM_CC_FAT | organelle membrane | RT | 5 | 9.6E-1 | 1.0E0 |
| **Annotation Cluster 39** | **Enrichment Score: 0.14** | | **Count** | **P_Value** | **Benjamini** |
| SP_PIR_KEYWORDS | iron | RT | 3 | 4.4E-1 | 9.4E-1 |
| GOTERM_MF_FAT | iron ion binding | RT | 3 | 6.4E-1 | 1.0E0 |
| SP_PIR_KEYWORDS | metal-binding | RT | 7 | 1.0E0 | 1.0E0 |
| GOTERM_MF_FAT | transition metal ion binding | RT | 8 | 1.0E0 | 1.0E0 |
| **Annotation Cluster 40** | **Enrichment Score: 0.11** | | **Count** | **P_Value** | **Benjamini** |
| GOTERM_BP_FAT | gamete generation | RT | 3 | 7.1E-1 | 1.0E0 |
| GOTERM_BP_FAT | sexual reproduction | RT | 3 | 7.9E-1 | 1.0E0 |
| GOTERM_BP_FAT | reproductive process in a multicellular organism | RT | 3 | 8.2E-1 | 1.0E0 |
| GOTERM_BP_FAT | multicellular organism reproduction | RT | 3 | 8.2E-1 | 1.0E0 |
| **Annotation Cluster 41** | **Enrichment Score: 0.09** | | **Count** | **P_Value** | **Benjamini** |
| GOTERM_BP_FAT | macromolecular complex assembly | RT | 4 | 8.0E-1 | 1.0E0 |
| GOTERM_BP_FAT | macromolecular complex subunit organization | RT | 4 | 8.3E-1 | 1.0E0 |
| GOTERM_BP_FAT | protein complex assembly | RT | 3 | 8.3E-1 | 1.0E0 |
| GOTERM_BP_FAT | protein complex biogenesis | RT | 3 | 8.3E-1 | 1.0E0 |

| Category | Term | | | | Count | P_Value | Benjamini |
|---|---|---|---|---|---|---|---|
| GOTERM_BP_FAT | protein complex biogenesis | RT | | | 3 | 8.3E-1 | 1.0E0 |
| **Annotation Cluster 42** | **Enrichment Score: 0.02** | | | | **Count** | **P_Value** | **Benjamini** |
| GOTERM_BP_FAT | cognition | RT | | | 4 | 9.3E-1 | 1.0E0 |
| GOTERM_BP_FAT | neurological system process | RT | | | 5 | 9.5E-1 | 1.0E0 |
| GOTERM_BP_FAT | sensory perception | RT | | | 3 | 9.7E-1 | 1.0E0 |
| **Annotation Cluster 43** | **Enrichment Score: 0.01** | | | | **Count** | **P_Value** | **Benjamini** |
| GOTERM_BP_FAT | phosphorylation | RT | | | 3 | 9.6E-1 | 1.0E0 |
| GOTERM_BP_FAT | phosphorus metabolic process | RT | | | 3 | 9.9E-1 | 1.0E0 |
| GOTERM_BP_FAT | phosphate metabolic process | RT | | | 3 | 9.9E-1 | 1.0E0 |
| **Annotation Cluster 44** | **Enrichment Score: 0** | | | | **Count** | **P_Value** | **Benjamini** |
| UP_SEQ_FEATURE | transmembrane region | RT | | | 3 | 1.0E0 | 1.0E0 |
| SP_PIR_KEYWORDS | transmembrane | RT | | | 3 | 1.0E0 | 1.0E0 |
| GOTERM_CC_FAT | integral to membrane | RT | | | 7 | 1.0E0 | 1.0E0 |

87 terms were not clustered.

Please cite *Nature Protocols 2009; 4(1):44 & Genome Biology 2003; 4(5):P3* within any publication that makes use of any methods inspired by **DAVID**.

Term of Service | Contact Us | Site Map

# Functional Annotation Clustering

Help and Manual

**Current Gene List: Primed secretomes**
**Current Background: Homo sapiens**
**83 DAVID IDs**

Options    Classification Stringency  Medium

Rerun using options    Create Sublist

**29 Cluster(s)**    Download File

| | | | | Count | P_Value | Benjamini |
|---|---|---|---|---|---|---|
| **Annotation Cluster 1** | **Enrichment Score: 3.76** | | | **Count** | **P_Value** | **Benjamini** |
| SP_PIR_KEYWORDS | acetylation | RT | | 24 | 1.2E-7 | 2.0E-5 |
| GOTERM_CC_FAT | cytoskeleton | RT | | 15 | 3.9E-4 | 1.0E-2 |
| GOTERM_CC_FAT | intracellular non-membrane-bounded organelle | RT | | 19 | 4.4E-3 | 6.7E-2 |
| GOTERM_CC_FAT | non-membrane-bounded organelle | RT | | 19 | 4.4E-3 | 6.7E-2 |
| **Annotation Cluster 2** | **Enrichment Score: 3.46** | | | **Count** | **P_Value** | **Benjamini** |
| GOTERM_CC_FAT | extracellular region | RT | | 21 | 1.5E-5 | 2.3E-3 |
| SP_PIR_KEYWORDS | signal | RT | | 23 | 2.0E-5 | 1.1E-3 |
| UP_SEQ_FEATURE | signal peptide | RT | | 23 | 2.2E-5 | 5.2E-3 |
| SP_PIR_KEYWORDS | Secreted | RT | | 16 | 3.4E-5 | 1.4E-3 |
| GOTERM_CC_FAT | extracellular region part | RT | | 14 | 3.6E-5 | 1.9E-3 |
| SP_PIR_KEYWORDS | glycoprotein | RT | | 22 | 3.7E-3 | 4.9E-2 |
| SP_PIR_KEYWORDS | disulfide bond | RT | | 16 | 1.1E-2 | 9.3E-2 |
| UP_SEQ_FEATURE | glycosylation site:N-linked (GlcNAc...) | RT | | 20 | 1.2E-2 | 7.6E-1 |
| UP_SEQ_FEATURE | disulfide bond | RT | | 15 | 1.9E-2 | 7.7E-1 |
| **Annotation Cluster 3** | **Enrichment Score: 3.1** | | | **Count** | **P_Value** | **Benjamini** |
| SP_PIR_KEYWORDS | extracellular matrix | RT | | 9 | 2.9E-7 | 2.4E-5 |
| GOTERM_CC_FAT | proteinaceous extracellular matrix | RT | | 9 | 2.2E-5 | 1.7E-3 |
| GOTERM_CC_FAT | extracellular matrix | RT | | 9 | 3.8E-5 | 1.5E-3 |
| SP_PIR_KEYWORDS | trimer | RT | | 4 | 4.9E-5 | 1.6E-3 |
| GOTERM_BP_FAT | collagen metabolic process | RT | | 4 | 1.3E-4 | 7.9E-2 |
| SP_PIR_KEYWORDS | collagen | RT | | 5 | 1.4E-4 | 3.7E-3 |
| GOTERM_BP_FAT | multicellular organismal macromolecule metabolic process | RT | | 4 | 1.8E-4 | 5.4E-2 |
| GOTERM_CC_FAT | collagen | RT | | 4 | 3.0E-4 | 9.3E-3 |
| GOTERM_BP_FAT | multicellular organismal metabolic process | RT | | 4 | 3.0E-4 | 6.1E-2 |
| SP_PIR_KEYWORDS | Ehlers-Danlos syndrome | RT | | 3 | 4.0E-4 | 8.3E-3 |
| GOTERM_CC_FAT | fibrillar collagen | RT | | 3 | 8.9E-4 | 2.0E-2 |
| GOTERM_CC_FAT | extracellular matrix part | RT | | 5 | 9.5E-4 | 1.8E-2 |
| INTERPRO | Collagen triple helix repeat | RT | | 4 | 2.2E-3 | 1.2E-1 |
| SP_PIR_KEYWORDS | triple helix | RT | | 3 | 3.3E-3 | 4.8E-2 |
| SP_PIR_KEYWORDS | hydroxylysine | RT | | 3 | 3.3E-3 | 4.8E-2 |
| GOTERM_MF_FAT | extracellular matrix structural constituent | RT | | 4 | 3.5E-3 | 1.2E-1 |
| SP_PIR_KEYWORDS | hydroxyproline | RT | | 3 | 4.7E-3 | 5.8E-2 |
| GOTERM_BP_FAT | collagen fibril organization | RT | | 3 | 4.7E-3 | 3.1E-1 |
| GOTERM_BP_FAT | extracellular matrix organization | RT | | 4 | 5.9E-3 | 3.4E-1 |
| KEGG_PATHWAY | ECM-receptor interaction | RT | | 4 | 6.1E-3 | 9.3E-2 |
| SP_PIR_KEYWORDS | hydroxylation | RT | | 3 | 1.6E-2 | 1.3E-1 |
| GOTERM_BP_FAT | extracellular structure organization | RT | | 4 | 2.0E-2 | 6.0E-1 |
| SP_PIR_KEYWORDS | coiled coil | RT | | 9 | 1.9E-1 | 6.5E-1 |
| **Annotation Cluster 4** | **Enrichment Score: 3.06** | | | **Count** | **P_Value** | **Benjamini** |
| SP_PIR_KEYWORDS | trimer | RT | | 4 | 4.9E-5 | 1.6E-3 |
| UP_SEQ_FEATURE | domain:TSP N-terminal | RT | | 3 | 1.8E-3 | 2.5E-1 |
| INTERPRO | Laminin G, thrombospondin-type, N-terminal | RT | | 3 | 2.0E-3 | 1.6E-1 |
| SMART | TSPN | RT | | 3 | 3.2E-3 | 6.3E-2 |
| **Annotation Cluster 5** | **Enrichment Score: 2.65** | | | **Count** | **P_Value** | **Benjamini** |

| Category | Term | RT | Count | P_Value | Benjamini |
|---|---|---|---|---|---|
| UP_SEQ_FEATURE | domain:Hemopexin-like 2 | RT | 3 | 1.8E-3 | 2.5E-1 |
| UP_SEQ_FEATURE | domain:Hemopexin-like 1 | RT | 3 | 1.8E-3 | 2.5E-1 |
| INTERPRO | Hemopexin/matrixin, repeat | RT | 3 | 2.2E-3 | 9.2E-2 |
| INTERPRO | Hemopexin/matrixin, conserved site | RT | 3 | 2.2E-3 | 9.2E-2 |
| INTERPRO | Hemopexin/matrixin | RT | 3 | 2.2E-3 | 9.2E-2 |
| SMART | HX | RT | 3 | 3.5E-3 | 4.6E-2 |
| **Annotation Cluster 6** | **Enrichment Score: 2.19** | | **Count** | **P_Value** | **Benjamini** |
| GOTERM_MF_FAT | polysaccharide binding | RT | 5 | 2.1E-3 | 9.4E-2 |
| GOTERM_MF_FAT | pattern binding | RT | 5 | 2.1E-3 | 9.4E-2 |
| GOTERM_MF_FAT | heparin binding | RT | 4 | 5.7E-3 | 1.5E-1 |
| KEGG_PATHWAY | ECM-receptor interaction | RT | 4 | 6.1E-3 | 9.3E-2 |
| GOTERM_MF_FAT | glycosaminoglycan binding | RT | 4 | 1.3E-2 | 2.7E-1 |
| GOTERM_MF_FAT | carbohydrate binding | RT | 5 | 3.6E-2 | 3.8E-1 |
| **Annotation Cluster 7** | **Enrichment Score: 2.14** | | **Count** | **P_Value** | **Benjamini** |
| UP_SEQ_FEATURE | short sequence motif:Cell attachment site | RT | 6 | 4.4E-6 | 2.1E-3 |
| GOTERM_BP_FAT | cell adhesion | RT | 9 | 3.0E-3 | 3.1E-1 |
| GOTERM_BP_FAT | biological adhesion | RT | 9 | 3.0E-3 | 2.7E-1 |
| SP_PIR_KEYWORDS | cell adhesion | RT | 6 | 6.0E-3 | 6.8E-2 |
| INTERPRO | EGF-like, type 3 | RT | 4 | 2.2E-2 | 5.3E-1 |
| INTERPRO | EGF-like | RT | 4 | 2.4E-2 | 5.0E-1 |
| SP_PIR_KEYWORDS | egf-like domain | RT | 4 | 2.6E-2 | 1.8E-1 |
| SMART | EGF | RT | 4 | 4.2E-2 | 3.5E-1 |
| INTERPRO | EGF | RT | 3 | 5.8E-2 | 7.2E-1 |
| INTERPRO | EGF-like region, conserved site | RT | 4 | 6.0E-2 | 7.0E-1 |
| **Annotation Cluster 8** | **Enrichment Score: 2.08** | | **Count** | **P_Value** | **Benjamini** |
| KEGG_PATHWAY | Focal adhesion | RT | 8 | 1.9E-5 | 6.2E-4 |
| GOTERM_BP_FAT | actin filament-based process | RT | 6 | 1.6E-3 | 2.2E-1 |
| SP_PIR_KEYWORDS | cytoskeleton | RT | 7 | 7.8E-3 | 7.4E-2 |
| GOTERM_BP_FAT | actin cytoskeleton organization | RT | 5 | 8.3E-3 | 4.1E-1 |
| GOTERM_BP_FAT | maintenance of protein location in cell | RT | 3 | 9.3E-3 | 4.2E-1 |
| GOTERM_BP_FAT | maintenance of location in cell | RT | 3 | 1.3E-2 | 4.9E-1 |
| GOTERM_BP_FAT | maintenance of protein location | RT | 3 | 1.3E-2 | 4.9E-1 |
| GOTERM_BP_FAT | cytoskeleton organization | RT | 6 | 1.9E-2 | 6.0E-1 |
| GOTERM_BP_FAT | maintenance of location | RT | 3 | 2.2E-2 | 6.0E-1 |
| GOTERM_MF_FAT | actin binding | RT | 5 | 2.8E-2 | 3.9E-1 |
| SP_PIR_KEYWORDS | actin-binding | RT | 4 | 3.1E-2 | 1.9E-1 |
| GOTERM_MF_FAT | cytoskeletal protein binding | RT | 5 | 1.0E-1 | 6.6E-1 |
| **Annotation Cluster 9** | **Enrichment Score: 1.45** | | **Count** | **P_Value** | **Benjamini** |
| GOTERM_MF_FAT | calcium ion binding | RT | 12 | 2.8E-4 | 2.0E-2 |
| SP_PIR_KEYWORDS | calcium | RT | 10 | 3.1E-4 | 7.4E-3 |
| GOTERM_MF_FAT | metal ion binding | RT | 13 | 8.4E-1 | 1.0E0 |
| GOTERM_MF_FAT | cation binding | RT | 13 | 8.5E-1 | 1.0E0 |
| GOTERM_MF_FAT | ion binding | RT | 13 | 8.7E-1 | 1.0E0 |
| **Annotation Cluster 10** | **Enrichment Score: 1.37** | | **Count** | **P_Value** | **Benjamini** |
| UP_SEQ_FEATURE | domain:EF-hand 2 | RT | 4 | 1.4E-2 | 7.3E-1 |
| UP_SEQ_FEATURE | domain:EF-hand 1 | RT | 4 | 1.4E-2 | 7.3E-1 |
| INTERPRO | EF-HAND 2 | RT | 4 | 2.8E-2 | 5.0E-1 |
| INTERPRO | Calcium-binding EF-hand | RT | 3 | 7.1E-2 | 7.2E-1 |
| SMART | EFh | RT | 3 | 1.0E-1 | 6.0E-1 |
| INTERPRO | EF-HAND 1 | RT | 3 | 1.4E-1 | 9.1E-1 |
| **Annotation Cluster 11** | **Enrichment Score: 1.33** | | **Count** | **P_Value** | **Benjamini** |
| GOTERM_BP_FAT | epidermis development | RT | 4 | 2.7E-2 | 6.5E-1 |
| SP_PIR_KEYWORDS | disease mutation | RT | 10 | 2.9E-2 | 1.9E-1 |
| GOTERM_BP_FAT | ectoderm development | RT | 4 | 3.3E-2 | 6.8E-1 |
| SP_PIR_KEYWORDS | coiled coil | RT | 9 | 1.9E-1 | 6.5E-1 |
| **Annotation Cluster 12** | **Enrichment Score: 1** | | **Count** | **P_Value** | **Benjamini** |
| GOTERM_CC_FAT | pigment granule | RT | 3 | 4.4E-2 | 3.9E-1 |
| GOTERM_CC_FAT | melanosome | RT | 3 | 4.4E-2 | 3.9E-1 |
| GOTERM_CC_FAT | | | | | |

| Category | Term | | Count | P_Value | Benjamini |
|---|---|---|---|---|---|
| | cytoplasmic membrane-bounded vesicle | RT | 6 | 5.4E-2 | 4.4E-1 |
| GOTERM_CC_FAT | membrane-bounded vesicle | RT | 6 | 6.1E-2 | 4.6E-1 |
| GOTERM_CC_FAT | cytoplasmic vesicle | RT | 6 | 9.2E-2 | 5.7E-1 |
| GOTERM_CC_FAT | vesicle | RT | 6 | 1.1E-1 | 6.0E-1 |
| GOTERM_CC_FAT | secretory granule | RT | 3 | 1.5E-1 | 6.9E-1 |
| GOTERM_CC_FAT | cytoplasmic vesicle part | RT | 3 | 1.6E-1 | 6.8E-1 |
| GOTERM_CC_FAT | mitochondrion | RT | 4 | 7.9E-1 | 1.0E0 |
| **Annotation Cluster 13** | **Enrichment Score: 0.97** | | **Count** | **P_Value** | **Benjamini** |
| GOTERM_MF_FAT | RNA binding | RT | 7 | 4.0E-2 | 3.8E-1 |
| SP_PIR_KEYWORDS | protein biosynthesis | RT | 3 | 9.5E-2 | 4.4E-1 |
| GOTERM_BP_FAT | translation | RT | 3 | 3.3E-1 | 9.9E-1 |
| **Annotation Cluster 14** | **Enrichment Score: 0.91** | | **Count** | **P_Value** | **Benjamini** |
| GOTERM_BP_FAT | negative regulation of protein metabolic process | RT | 4 | 2.9E-2 | 6.4E-1 |
| GOTERM_BP_FAT | negative regulation of cellular protein metabolic process | RT | 3 | 1.3E-1 | 9.5E-1 |
| GOTERM_BP_FAT | regulation of cellular protein metabolic process | RT | 4 | 2.4E-1 | 9.9E-1 |
| GOTERM_BP_FAT | negative regulation of macromolecule metabolic process | RT | 5 | 2.6E-1 | 9.9E-1 |
| **Annotation Cluster 15** | **Enrichment Score: 0.82** | | **Count** | **P_Value** | **Benjamini** |
| GOTERM_MF_FAT | protein kinase binding | RT | 3 | 9.5E-2 | 6.6E-1 |
| GOTERM_MF_FAT | kinase binding | RT | 3 | 1.3E-1 | 7.1E-1 |
| GOTERM_MF_FAT | enzyme binding | RT | 4 | 2.8E-1 | 8.9E-1 |
| **Annotation Cluster 16** | **Enrichment Score: 0.77** | | **Count** | **P_Value** | **Benjamini** |
| SP_PIR_KEYWORDS | zymogen | RT | 4 | 1.9E-2 | 1.4E-1 |
| GOTERM_CC_FAT | extracellular space | RT | 7 | 4.2E-2 | 4.0E-1 |
| SP_PIR_KEYWORDS | metal-binding | RT | 4 | 9.9E-1 | 1.0E0 |
| GOTERM_MF_FAT | transition metal ion binding | RT | 4 | 1.0E0 | 1.0E0 |
| **Annotation Cluster 17** | **Enrichment Score: 0.77** | | **Count** | **P_Value** | **Benjamini** |
| GOTERM_BP_FAT | macromolecular complex assembly | RT | 6 | 8.5E-2 | 9.2E-1 |
| GOTERM_BP_FAT | protein complex biogenesis | RT | 5 | 1.0E-1 | 9.4E-1 |
| GOTERM_BP_FAT | protein complex assembly | RT | 5 | 1.0E-1 | 9.4E-1 |
| GOTERM_BP_FAT | macromolecular complex subunit organization | RT | 6 | 1.1E-1 | 9.3E-1 |
| GOTERM_BP_FAT | cellular macromolecular complex subunit organization | RT | 4 | 1.3E-1 | 9.6E-1 |
| GOTERM_BP_FAT | cellular macromolecular complex assembly | RT | 3 | 3.1E-1 | 1.0E0 |
| GOTERM_BP_FAT | regulation of transcription | RT | 3 | 1.0E0 | 1.0E0 |
| **Annotation Cluster 18** | **Enrichment Score: 0.69** | | **Count** | **P_Value** | **Benjamini** |
| GOTERM_CC_FAT | extracellular space | RT | 7 | 4.2E-2 | 4.0E-1 |
| GOTERM_BP_FAT | response to endogenous stimulus | RT | 3 | 4.2E-1 | 1.0E0 |
| GOTERM_BP_FAT | response to organic substance | RT | 4 | 4.8E-1 | 1.0E0 |
| **Annotation Cluster 19** | **Enrichment Score: 0.68** | | **Count** | **P_Value** | **Benjamini** |
| GOTERM_BP_FAT | sexual reproduction | RT | 5 | 7.9E-2 | 9.2E-1 |
| GOTERM_BP_FAT | gamete generation | RT | 4 | 1.6E-1 | 9.7E-1 |
| GOTERM_BP_FAT | reproductive developmental process | RT | 3 | 2.4E-1 | 9.9E-1 |
| GOTERM_BP_FAT | multicellular organism reproduction | RT | 4 | 2.5E-1 | 9.9E-1 |
| GOTERM_BP_FAT | reproductive process in a multicellular organism | RT | 4 | 2.5E-1 | 9.9E-1 |
| GOTERM_BP_FAT | spermatogenesis | RT | 3 | 3.0E-1 | 9.9E-1 |
| GOTERM_BP_FAT | male gamete generation | RT | 3 | 3.0E-1 | 9.9E-1 |
| **Annotation Cluster 20** | **Enrichment Score: 0.66** | | **Count** | **P_Value** | **Benjamini** |
| GOTERM_BP_FAT | Ras protein signal transduction | RT | 3 | 5.3E-2 | 8.3E-1 |
| GOTERM_BP_FAT | small GTPase mediated signal transduction | RT | 3 | 2.9E-1 | 9.9E-1 |
| GOTERM_BP_FAT | intracellular signaling cascade | RT | 5 | 6.6E-1 | 1.0E0 |
| **Annotation Cluster 21** | **Enrichment Score: 0.64** | | **Count** | **P_Value** | **Benjamini** |
| GOTERM_BP_FAT | positive regulation of cellular component organization | RT | 5 | 3.8E-3 | 2.9E-1 |
| GOTERM_CC_FAT | internal side of plasma membrane | RT | 4 | 1.2E-1 | 6.1E-1 |
| GOTERM_CC_FAT | plasma membrane part | RT | 9 | 6.0E-1 | 9.9E-1 |
| GOTERM_CC_FAT | plasma membrane | RT | 14 | 7.0E-1 | 9.9E-1 |
| SP_PIR_KEYWORDS | cell membrane | RT | 6 | 7.4E-1 | 1.0E0 |
| SP_PIR_KEYWORDS | membrane | RT | 10 | 1.0E0 | 1.0E0 |

| | | | | | | |
|---|---|---|---|---|---|---|
| **Annotation Cluster 22** | **Enrichment Score: 0.63** | | | **Count** | **P_Value** | **Benjamini** |
| UP_SEQ_FEATURE | nucleotide phosphate-binding region:GTP | RT | | 4 | 4.8E-2 | 9.6E-1 |
| SP_PIR_KEYWORDS | gtp-binding | RT | | 4 | 6.2E-2 | 3.2E-1 |
| GOTERM_MF_FAT | GTP binding | RT | | 4 | 1.4E-1 | 7.3E-1 |
| GOTERM_MF_FAT | guanyl ribonucleotide binding | RT | | 4 | 1.5E-1 | 7.3E-1 |
| GOTERM_MF_FAT | guanyl nucleotide binding | RT | | 4 | 1.5E-1 | 7.3E-1 |
| GOTERM_MF_FAT | GTPase activity | RT | | 3 | 1.7E-1 | 7.5E-1 |
| SP_PIR_KEYWORDS | nucleotide-binding | RT | | 6 | 5.0E-1 | 9.6E-1 |
| GOTERM_MF_FAT | purine nucleotide binding | RT | | 8 | 5.3E-1 | 9.9E-1 |
| GOTERM_CC_FAT | microtubule cytoskeleton | RT | | 3 | 6.2E-1 | 9.9E-1 |
| GOTERM_MF_FAT | purine ribonucleotide binding | RT | | 7 | 6.5E-1 | 1.0E0 |
| GOTERM_MF_FAT | ribonucleotide binding | RT | | 7 | 6.5E-1 | 1.0E0 |
| **Annotation Cluster 23** | **Enrichment Score: 0.54** | | | **Count** | **P_Value** | **Benjamini** |
| GOTERM_MF_FAT | RNA binding | RT | | 7 | 4.0E-2 | 3.8E-1 |
| SP_PIR_KEYWORDS | rna-binding | RT | | 5 | 6.1E-2 | 3.3E-1 |
| SP_PIR_KEYWORDS | ribonucleoprotein | RT | | 3 | 1.8E-1 | 6.4E-1 |
| GOTERM_CC_FAT | organelle lumen | RT | | 10 | 2.4E-1 | 8.2E-1 |
| GOTERM_CC_FAT | membrane-enclosed lumen | RT | | 10 | 2.6E-1 | 8.3E-1 |
| GOTERM_CC_FAT | nucleolus | RT | | 5 | 2.7E-1 | 8.2E-1 |
| GOTERM_CC_FAT | ribonucleoprotein complex | RT | | 4 | 3.0E-1 | 8.5E-1 |
| GOTERM_CC_FAT | intracellular organelle lumen | RT | | 8 | 5.1E-1 | 9.7E-1 |
| GOTERM_BP_FAT | RNA processing | RT | | 3 | 5.8E-1 | 1.0E0 |
| GOTERM_CC_FAT | nuclear lumen | RT | | 6 | 6.5E-1 | 9.9E-1 |
| GOTERM_CC_FAT | nucleoplasm | RT | | 3 | 8.5E-1 | 1.0E0 |
| SP_PIR_KEYWORDS | nucleus | RT | | 9 | 9.3E-1 | 1.0E0 |
| **Annotation Cluster 24** | **Enrichment Score: 0.52** | | | **Count** | **P_Value** | **Benjamini** |
| GOTERM_BP_FAT | intracellular transport | RT | | 6 | 8.2E-2 | 9.2E-1 |
| GOTERM_BP_FAT | protein targeting | RT | | 3 | 1.8E-1 | 9.8E-1 |
| GOTERM_BP_FAT | cellular protein localization | RT | | 4 | 1.8E-1 | 9.7E-1 |
| GOTERM_BP_FAT | cellular macromolecule localization | RT | | 4 | 1.8E-1 | 9.7E-1 |
| GOTERM_BP_FAT | protein localization | RT | | 6 | 2.0E-1 | 9.8E-1 |
| GOTERM_BP_FAT | induction of apoptosis | RT | | 3 | 3.1E-1 | 9.9E-1 |
| GOTERM_BP_FAT | induction of programmed cell death | RT | | 3 | 3.2E-1 | 9.9E-1 |
| GOTERM_BP_FAT | regulation of apoptosis | RT | | 5 | 3.2E-1 | 9.9E-1 |
| GOTERM_BP_FAT | regulation of programmed cell death | RT | | 5 | 3.3E-1 | 9.9E-1 |
| GOTERM_BP_FAT | regulation of cell death | RT | | 5 | 3.3E-1 | 9.9E-1 |
| GOTERM_BP_FAT | intracellular protein transport | RT | | 3 | 3.8E-1 | 1.0E0 |
| GOTERM_BP_FAT | positive regulation of apoptosis | RT | | 3 | 4.5E-1 | 1.0E0 |
| GOTERM_BP_FAT | positive regulation of programmed cell death | RT | | 3 | 4.6E-1 | 1.0E0 |
| GOTERM_BP_FAT | positive regulation of cell death | RT | | 3 | 4.6E-1 | 1.0E0 |
| GOTERM_BP_FAT | establishment of protein localization | RT | | 4 | 5.2E-1 | 1.0E0 |
| GOTERM_BP_FAT | protein transport | RT | | 3 | 7.6E-1 | 1.0E0 |
| **Annotation Cluster 25** | **Enrichment Score: 0.5** | | | **Count** | **P_Value** | **Benjamini** |
| GOTERM_CC_FAT | synapse part | RT | | 3 | 2.4E-1 | 8.3E-1 |
| UP_SEQ_FEATURE | mutagenesis site | RT | | 8 | 3.4E-1 | 1.0E0 |
| GOTERM_CC_FAT | synapse | RT | | 3 | 3.9E-1 | 9.1E-1 |
| **Annotation Cluster 26** | **Enrichment Score: 0.46** | | | **Count** | **P_Value** | **Benjamini** |
| SP_PIR_KEYWORDS | zymogen | RT | | 4 | 1.9E-2 | 1.4E-1 |
| GOTERM_MF_FAT | endopeptidase activity | RT | | 3 | 3.9E-1 | 9.6E-1 |
| SP_PIR_KEYWORDS | Protease | RT | | 3 | 3.9E-1 | 9.1E-1 |
| GOTERM_MF_FAT | peptidase activity, acting on L-amino acid peptides | RT | | 3 | 5.9E-1 | 9.9E-1 |
| GOTERM_MF_FAT | peptidase activity | RT | | 3 | 6.1E-1 | 9.9E-1 |
| GOTERM_BP_FAT | proteolysis | RT | | 4 | 7.3E-1 | 1.0E0 |
| SP_PIR_KEYWORDS | hydrolase | RT | | 4 | 8.1E-1 | 1.0E0 |
| **Annotation Cluster 27** | **Enrichment Score: 0.16** | | | **Count** | **P_Value** | **Benjamini** |
| GOTERM_MF_FAT | nucleotide binding | RT | | 11 | 2.6E-1 | 8.9E-1 |
| SP_PIR_KEYWORDS | nucleotide-binding | RT | | 6 | 5.0E-1 | 9.6E-1 |
| GOTERM_MF_FAT | purine nucleotide binding | RT | | 8 | 5.3E-1 | 9.9E-1 |

| Category | Term | | Count | P_Value | Benjamini |
|---|---|---|---|---|---|
| GOTERM_MF_FAT | purine ribonucleotide binding | RT | 7 | 6.5E-1 | 1.0E0 |
| GOTERM_MF_FAT | ribonucleotide binding | RT | 7 | 6.5E-1 | 1.0E0 |
| GOTERM_BP_FAT | cell cycle | RT | 3 | 7.7E-1 | 1.0E0 |
| GOTERM_MF_FAT | adenyl nucleotide binding | RT | 4 | 9.3E-1 | 1.0E0 |
| GOTERM_MF_FAT | purine nucleoside binding | RT | 4 | 9.3E-1 | 1.0E0 |
| GOTERM_MF_FAT | nucleoside binding | RT | 4 | 9.4E-1 | 1.0E0 |
| GOTERM_MF_FAT | ATP binding | RT | 3 | 9.7E-1 | 1.0E0 |
| GOTERM_MF_FAT | adenyl ribonucleotide binding | RT | 3 | 9.8E-1 | 1.0E0 |
| **Annotation Cluster 28** | **Enrichment Score: 0.09** | | **Count** | **P_Value** | **Benjamini** |
| GOTERM_CC_FAT | cell fraction | RT | 4 | 7.8E-1 | 1.0E0 |
| GOTERM_CC_FAT | membrane fraction | RT | 3 | 8.2E-1 | 1.0E0 |
| GOTERM_CC_FAT | insoluble fraction | RT | 3 | 8.3E-1 | 1.0E0 |
| **Annotation Cluster 29** | **Enrichment Score: 0** | | **Count** | **P_Value** | **Benjamini** |
| UP_SEQ_FEATURE | topological domain:Cytoplasmic | RT | 3 | 1.0E0 | 1.0E0 |
| UP_SEQ_FEATURE | transmembrane region | RT | 3 | 1.0E0 | 1.0E0 |
| SP_PIR_KEYWORDS | transmembrane | RT | 3 | 1.0E0 | 1.0E0 |
| GOTERM_CC_FAT | intrinsic to membrane | RT | 6 | 1.0E0 | 1.0E0 |
| GOTERM_CC_FAT | integral to membrane | RT | 5 | 1.0E0 | 1.0E0 |

50 terms were not clustered.

---

Please cite *Nature Protocols 2009; 4(1):44 & Genome Biology 2003; 4(5):P3* within any publication that makes use of any methods inspired by **DAVID**.

SAIC Frederick · NIAID · ABCC · NCI-Frederick · National Institutes of Health · FirstGov · Department of Health and Human Services

Term of Service | Contact Us | Site Map
